# Predicting the potential distribution of *Dactylorhiza hatagirea* (D. Don) Soo-an important medicinal orchid in the West Himalaya, under multiple climate change scenarios

**DOI:** 10.1371/journal.pone.0269673

**Published:** 2022-06-17

**Authors:** Laxman Singh, Nidhi Kanwar, Indra D. Bhatt, Shyamal K. Nandi, Anil K. Bisht

**Affiliations:** 1 Center for Biodiversity Conservation and Management, G. B. Pant National Institute of Himalayan Environment, Kosi-Katarmal, Almora, Uttarakhand, India; 2 Center for Environment Assessment and Climate Change, G. B. Pant National Institute of Himalayan Environment, Kosi-Katarmal, Almora, Uttarakhand, India; 3 Department of Botany, D. S. B. Campus, Kumaun University, Nainital, Uttarakhand, India; Instituto Federal de Educacao Ciencia e Tecnologia Goiano - Campus Urutai, BRAZIL

## Abstract

Climate variability coupled with anthropogenic pressures is the most critical driver in the Himalayan region for forest ecosystem vulnerability. *Dactylorhiza hatagirea* (D.Don) Soo is an important yet highly threatened medicinal orchid from the Himalayan region. Poor regenerative power and growing demand have resulted in the steep decline of its natural habitats populations. The present study aims to identify the habitat suitability of *D*. *hatagirea* in the Western Himalaya using the maximum entropy model (MaxEnt). The community climate system model (CCSM ver. 4) based on representative concentration pathways (RCPs) was used to determine suitable future areas. Sixteen least correlated (< 0.8) bioclimatic, topographical and geomorphic variables were used to construct the species climatic niche. The dominant contributing variables were elevation (34.85%) followed by precipitation of the coldest quarter (23.04%), soil type (8.77%), land use land cover (8.26%), mean annual temperature (5.51%), and temperature seasonality (5.11%). Compared to the present distribution, habitat suitability under future projection, i.e., RCP 4.5 and RCP 8.5 (2050 and 2070), was found to shift to higher elevation towards the northwest direction, while lower altitudes will invariably be less suitable. Further, as compared to the current distribution, the climatic niche space of the species is expected to expand in between11.41–22.13% in the near future. High habitats suitability areas are mainly concentrated in the forest range like Dharchula and Munsyari range, Pindar valley, Kedarnath Wildlife Sanctuary, West of Nanda Devi Biosphere Reserve, and Uttarkashi forest division. The present study delineated the fundamental niche baseline map of *D*. *hatagirea* in the Western Himalayas and highlighted regions/areas where conservation and management strategies should be intensified in the next 50 years. In addition, as the species is commercially exploited illegally, the information gathered is essential for conservationists and planners who protect the species at the regional levels.

## 1. Introduction

Climate is an important ecological and abiotic factor affecting species potential geographic distribution and ecological niche space [1]. The minimal changes in species bioclimatic envelope are thought to have considerable impacts on the plant-pollinator relationship, seed set, and regeneration status. It is expected that species may no longer adapt to a set of environmental conditions to facilitate further expansion [2]. Therefore, the species must either cope with the prevailing ecological conditions or colonize to sustain or become extinct [3]. This has led to a growing interest in developing and scaling up prioritization strategies for such species to ensure the highest conservation gains [4].

The Himalayan regions are an assemblage of biodiversity hotspots [5]. However, the ongoing disturbance exacerbated by climate change, habitat fragmentation, invasions by alien species, grazing and trampling, overexploitation, and excessive consumption of natural resources has altered the structural and functional integrity of the various Himalayan ecosystems [6]. Besides these, climate variability, land-use change, and rural migration are key contributors to biodiversity loss in the region [7]. In the last few decades, the region saw cascading effects of climate variability mainly due to increased greenhouse gases concentration. It is believed that the rate of global warming in the Himalayas is much higher than the global average. For instance, in the last 100 years, the global average temperature rise was 0.74°C [8]. However, in the Himalayas, a 1.5°C temperature increase was documented in the final quarter (i.e., 1982–2006) of the twentieth century [9], with warming potentially reaching 5°C by the end of the twenty-first century [9, 10]. This rise is alarming because Himalayan floras are alienated to specific elevation gradient/microhabitat conditions [3, 11]. The shift in the climatic envelope is expected to bring significant change in the resident species habitat conditions, leading to changes in species richness, population structure, and those unable to cope are likely to face local extinction [1–3].

Furthermore, the recent upsurge in herbal or its derived products across the globe has led to uncontrolled abusive practice; thus, the natural stock of these plants is under tremendous pressure. In the case of the Indian Himalayan Region (IHR), a considerable number (1748 species) of medicinal and aromatic plants (MAPs) are reported, with 31% of them being native, whereas 15.5% are endemic and threatened [12]. The high potential instability and inherent vulnerability make the region one of the most ecologically fragile bio-geographic zones [13]. Other challenges on the MAPs include low population size, habitat specificity, genetic bottleneck effect, narrow distributional ranges, and heavy livestock grazing [14]. The literature on these threatened plants is fragmentary or limited to specific geographic pockets [15]. In the above context, it is obligatory to make a conservation framework encircling species habitat restoration and promote cultivation, thus, reducing pressure on the wild populations.

The development of statistical modelling and geospatial technology in predicting suitable habitat distribution has gained immense popularity. However, such information is at an initial phase for the Himalayan MAPs [6]. The use of geospatial technology could add an advantage as obtaining specific distribution maps for such species is difficult and often requires intensive surveys [16]. The difficulty level becomes amplified in the Himalayan region where the working conditions are not conducive for the survey, i.e., inaccessible and difficult topography perplexed with hostile conditions. Therefore, estimating current plants distribution and identifying important climatic refugia will help predict future distribution patterns and reveal regions with high extinction rates. At present, the common method to study potentially species distribution and environmentally suitable habitats is to use species distribution models (SDMs) [17]. SDM has made it possible to analyze the environmental drivers of species distributions and project a species realized niche into a geographic area [18]. Of many SDM algorithm methods, MaxEnt has proved decisive when modeling rare species with narrow ranges [19]. MaxEnt modelling is a robust computational algorithm that works on the backdrop of species presence points and rasterized environmental data. The probable ecological niche can be reconstructed using species presence data points and environmental variables/predictors [20]. Such model-based sampling would become an important benchmark for endemics and threatened species and is a well-recognized cost-efficient method [21].

**Fig 1:**
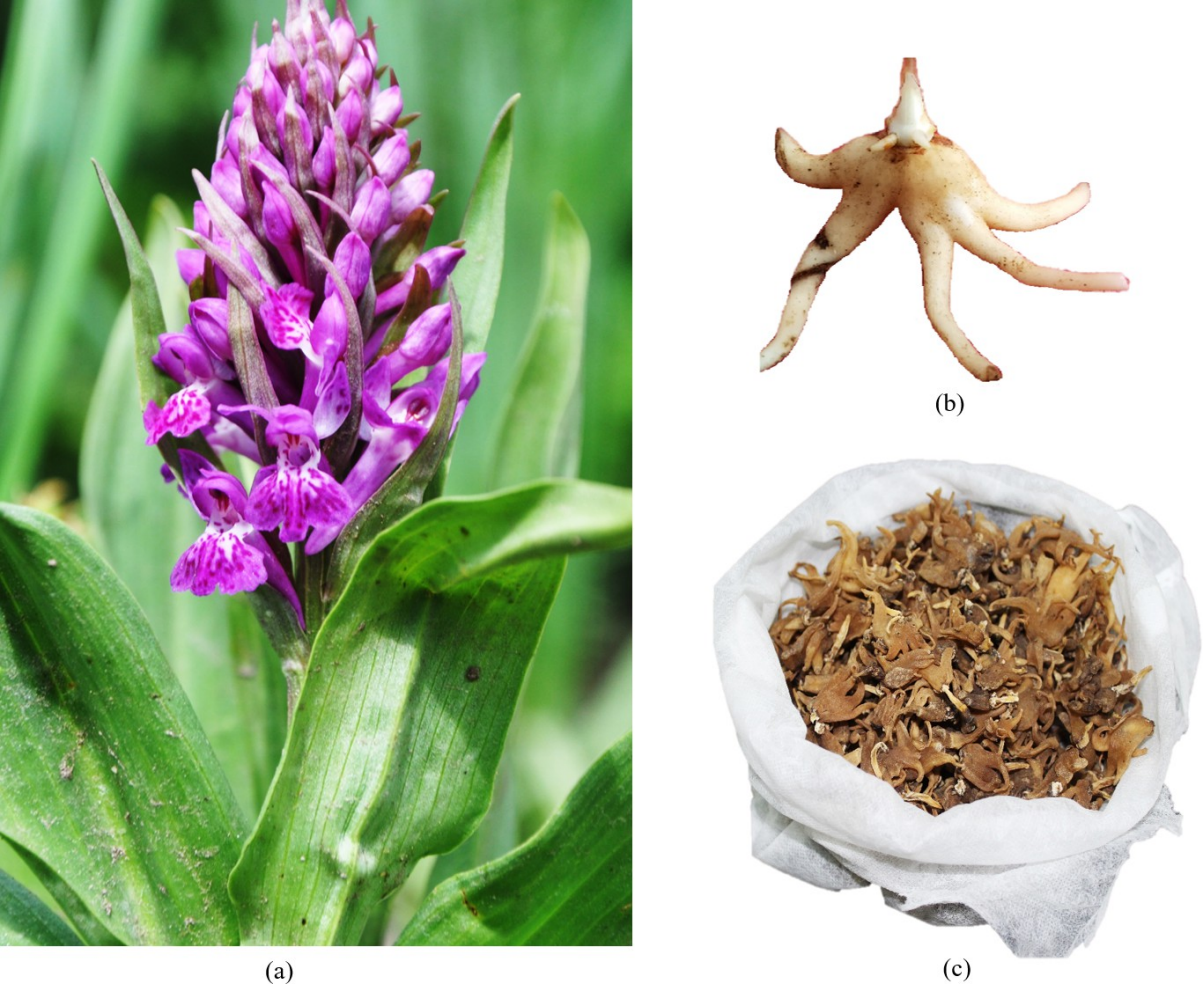
Image showing (a) compact floral structure of *D*. *hatagirea*, (b) well developed palmately lobed tuber, and (c) tuber collected and processed as a marketed product.

In the present study, an effort has been made to model the potential habitat distribution and effect of future climate change on *D*. *hatagirea*, a critically endangered [22, 23] and endemic species of the Himalayan region. The species is a tall, slender, ground-dwelling, perennial herb with palmately lobed tuberoids that prefer to grow in a moist, mild, acidic soil environment (Fig 1A and 1B). The species has been reported from India, Afghanistan, Pakistan, Nepal, Tibet, and Bhutan [6]. In the IHR, the species is reported from Jammu and Kashmir, Ladakh, Himachal Pradesh, Uttarakhand, Sikkim, and Arunachal Pradesh at an altitudinal range of 2500 to 4500 m above sea level (asl). The estimated annual trade of the species is around 10–50 metric tons [24], with an economic value of US $ 68.88–89.54 (1US $ = Rs. 77.39) kg^-1^ of the dried tuber (Fig 1C). At present, the tuber of the species is destructively harvested and illegally traded; thus, it puts a stake on its future existence. Moreover, the species require specific microhabitat conditions for growth and perturbation, thus limiting the species widespread distribution. Therefore, to minimize the pressure on the wild populations, efforts are ongoing to develop and upscale the existing multiplication strategies for mass multiplication. Meanwhile, mapping and conserving the critical habitat is expected to offer a possible solution to species conservation and management. The study attempts to address the following scientific questions: (i) What is the present potential geographical distribution range of *Dactylorhiza hatagirea* in the Western Himalaya, India? (ii) What will be the impacts of climate variability on the future distribution of *D*. *hatagirea* using four Representative Concentration Pathways (RCPs)?, and (iii) Where are the high potential distributional areas of *D*. *hatagirea* that could be protected or could be suggested for cultivation, reintroduction/ recovery plans?. Answering these questions will help identify suitable habitats for the conservation of the species, which may help policy planers while developing strategies for its conservation.

## 2. Materials and methods

### 2.1. Study area and ecological significance

**Fig 2:**
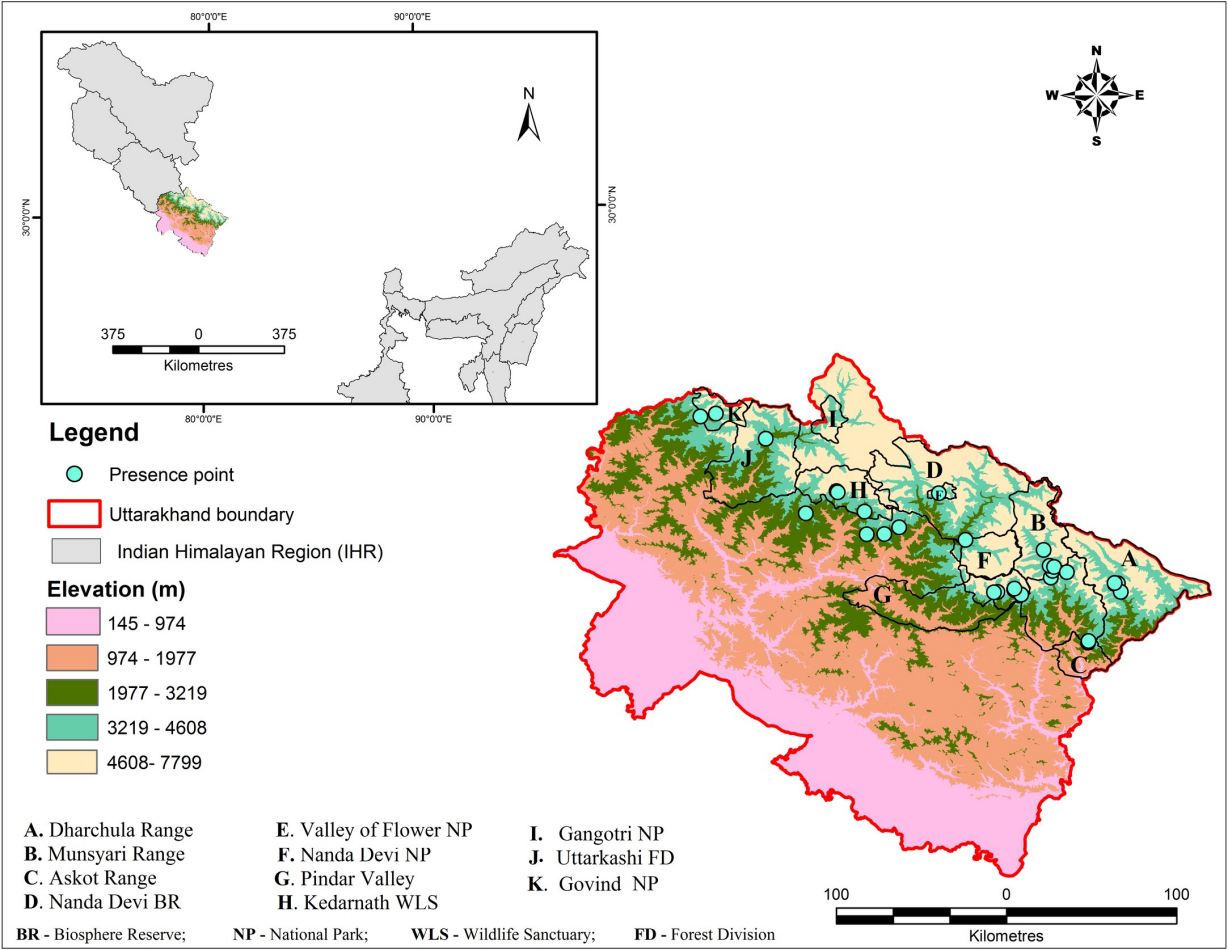
Field collected *D*. *hatagirea* (blue dots) points mounted on the elevation map of Uttarakhand state. Maps in Fig 1 are generated with ArcGIS version 10.3 (ESRI, CA, USA).

The study was undertaken in Uttarakhand state (28°43ˈ to 31°28ˈ N Latitude and 77°34ˈ to 80°03ˈ E Longitude) of IHR (Fig 2). The state has a total recorded forest area (RFA) of 38,000 km^2^ (71.05% of its total geographical area 53,485 km^2^), out of which 26,547 km^2^ is reserved forest, 9,885 km^2^ is protected forest, and 1,568 km^2^ is unclassed forests [25]. The state experience varied climates from warm dry to warm wet and with a latent cool, dry period. The state’s temperature ranges from sub-zero to 43°C [26], and average annual rainfall varies from 1093.8 mm to 1385.5 mm [27].

The present study area harbors alpine vegetation, which covers 8,524 km^2^. Of these, 4,376 km^2^ is surmounted by permanent snow cover (i.e., corresponding to ca.24.11% statealpine geographical area) [28]. The alpine regions are well known for their high-value MAPs, including *D*. *hatagirea*. The region is experiencing major environmental transformation repercussions, and anthropogenic activities outnumber the natural eventualities, thus enforcing species to various threat categories [15].

### 2.2. Species point data

To predict species distribution, it is a prerequisite to have species presence points and environmental variables [29]. The data search was primarily made from online portals such as Global Biodiversity Information Facility [30], published literature, and herbarium consultation Botanical Survey of India, Dehradun (BSID). Data on the species were very limited, whereas herbarium records were not geo-referenced. Considering these limitations, an extensive field survey was conducted during 2016–19, and presence points were recorded. A total of 30 occurrences of the species were recorded during field surveys. A portable multi-channel Global Positioning System (Garmin) receiver with 10–20 m positional accuracy was used to record the species occurrence geo-coordinates. The coordinates were then converted to decimal degrees and used to model the species potential habitats distribution in its native range.

### 2.3. Data source

The climate data were downloaded from World Climate Database [31]. WorldClim provides current (baseline) and projected climate data for 2070 with a spatial resolution of 30 seconds (ca. 1 km) in GeoTIFF format. These climatic data are derivatives of maximum, minimum, and average values of monthly, quarterly, and annual temperatures and precipitation of the last 30 years, i.e., 1970–2000. Likewise, environmental variables such as soil type, soil moisture, and soil pH were downloaded from the International Soil Reference Information Centre [32], while land use land cover (LULC) from http://www.esa-landcover-cci.org/ [33] (Table 1). Besides these, non-climatic variables, i.e., altitude, aspect derived from NASA Shuttle Radar Topographic Mission (SRTM, version 4.1) [34]. The reason behind using both the climatic and non-climatic variables is to enhance the model’s predictive power as suggested for endemic plants [5, 35]. Further, for future prediction studies, we used Community Climate System Model (CCSM) ver. 4 (CCSM4) that is based on the Fifth Assessment Report (AR 5) of the Intergovernmental Panel on Climate Change [36] and two contrasting Representative Concentrations Pathways (RCP 4.5 and RCP8.5) for the years 2050 and 2070. Furthermore, we assumed that the edaphic properties are expected to remain stable in the next several decades, as soil properties should not change synchronously with sudden climate change; hence the same raster layer was used in future projections.

**Table 1:** Environmental variables and their percent contributions for predicting the potential distribution of *D*. *hatagirea*.

Type	Code	Variable name	Unit	% Contribution
Climatic	Bio1	Annual mean temperature	°C	5.51
	Bio2	Mean diurnal range (mean of monthly max. and min. temp.)	°C	0.07
	Bio3	Isothermality [(Bio2/Bio7) x 100]	-	0.98
	Bio4	Temperature seasonality (standard deviation x 100)	C of V	5.11
	Bio7	Temperature annual range (Bio5-Bio6)	°C	1.48
	Bio8	Mean temperature of wettest quarter	°C	0.08
	Bio12	Annual precipitation	mm	1.95
	Bio18	Precipitation of warmest quarter	mm	4.38
	Bio19	Precipitation of coldest quarter	mm	23.04
Geomorphologic	DEM	DEM	°	34.85
	SLP	SLOPE	°	3.06
		ASPECT		0.98
Pedologic		SOIL TYPE		8.77
		SOIL MOISTURE	mm	1.13
		SOIL pH		0.36
Land use land cover		LULC		8.26

#### 2.3.1. Environmental layers and variable selection

The model’s output can be accurate, biologically meaningful, and generalized if built with predictor variables that directly impact species distribution. Strong collinearity between the variables in SDMs may cause model overfitting due to the high level of correlation among variables [37]. To avoid multi-collinearity among the 19 bioclimatic variables, highly correlated variables (r ≥ 0.80 Pearson correlation coefficient) were eliminated from further models using ENM Tools. This reduction of predictor variables resulted in the inclusion of nine bioclimatic variables and seven environmental variables for the prediction process (S1 Table). Further, using ArcGIS 10.3, all predictor variables layers were rasterized into the same bounds, cell sizes, and coordinate system as the layer of occurrence localities. Finally, these layers were converted to ASCII format for further processing in MaxEnt.

#### 2.3.2. Model parameterization

MaxEnt algorithm (MaxEnt ver. 3.4.1) [38] for habitat distribution modelling was employed [29]. MaxEnt algorithm was chosen over other available machine learning tools owing to (i) presence of only data points of the species, (ii) works even relatively with a small number of occurrence locations and high predictive performance, (iii) can handle continuous and categorical environmental data simultaneously, (iv) analyze results in terms of percent contribution of environmental data through model output, (v) examine variables weight through jackknife method, and (vi) calibrate the model, run numerous replicates along with cross-validation, and bootstrapping to test model robustness [18, 39, 40]. We used 75% of the dataset for training and 25% dataset model testing in this study. For generating model robustness, the number of iterations was set to 5000, with 30 replicated model runs. The maximum background points10000 and ten percentile training presence with logistic threshold rule were applied, whereas other parameters were set to default.

#### 2.3.3. Model performance and potential niche change

To calibrate the model and validate its robustness, threshold independent receiver-operating characteristic analysis (ROC) and area under the receiver-operating characteristic curve (AUC) were tested for model precision. The AUC value varies between 0 to1. The values close to +1 indicate conformity between observations and prediction, whereas zero or less values indicate a performance no better than random [41]. Statistically, AUC values near 1 indicate very good model performance, whereas AUC values close to 0 signify complete inaccurate prediction. Model performance based on AUC values are categorized as, very good (0.95 < AUC < 1.0), good (0.9 < AUC < 0.95), fair (0.8 < AUC < 0.9), and poor (AUC < 0.8) [42, 43]. In the past, several studies have suggested that the AUC values mislead the performance of predictive distribution models and reflect relative model performance [44]. Therefore, to assess the predictive success of models, sensitivity, specificity, overall accuracy, and True Skill Statistics (TSS) were calculated by a confusion matrix. Threshold-dependent TSS is considered an additional accuracy measurement that is not affected by prevalence as it does for the kappa coefficient and the size of the validation set [45]. It deals with sensitivity and specificity, values ranging from − 1 to + 1, where + 1 indicates perfect agreement, scores ranging from 0.6 to 0.9 specify fair to good model performance, and 0 represents a random fit [45]. For this, the output of the logistic layer derived from MaxEnt results was reclassified into a binary prediction map (unsuitable and suitable) with a threshold of 10 percentile training presence. All geographical plotting and suitable range-size estimation were conducted in ArcGIS software (version 10.3).

To identify the potential area of distribution, the distributional indices based on threshold interval classification (TIC) were categorized as highly suitable (TIC > 0.75), moderate suitable (0.50 < TIC < 0.75), least suitable (0.25 < TIC < 0.50), and unsuitable areas (TIC < 0.25). Changes in the potential niche of *D*. *hatagirea* between the current and future climatic scenarios were computed by converting ASCII output projections into raster format using ArcGIS 10.3. Simultaneously the number of cells (pixels) among the projected climatic extent was calculated using zonal statistics in spatial analyst tools in ArcGIS 10.3. The differences in the mean number of cells among four classes of potential habitats were converted to surface area (km^2^). Finally, MaxEnt predictive maps for the current and future scenarios were related to elevation classes. This would help map habitats and contribute to species-specific interventions/ reintroduction programs.

## 3. Results

### 3.1. Preliminary screening of model inputs variables

The credibility of any prediction model is dependent on input variables for species distribution modelling. Given this, sixteen predictor variables out of twenty-six variables; with correlation coefficients of ≤ 0.8 were retained after preliminary screening and selected for further modelling (Table 1).

### 3.2. Model performance and variable contributions

**Fig 3:**
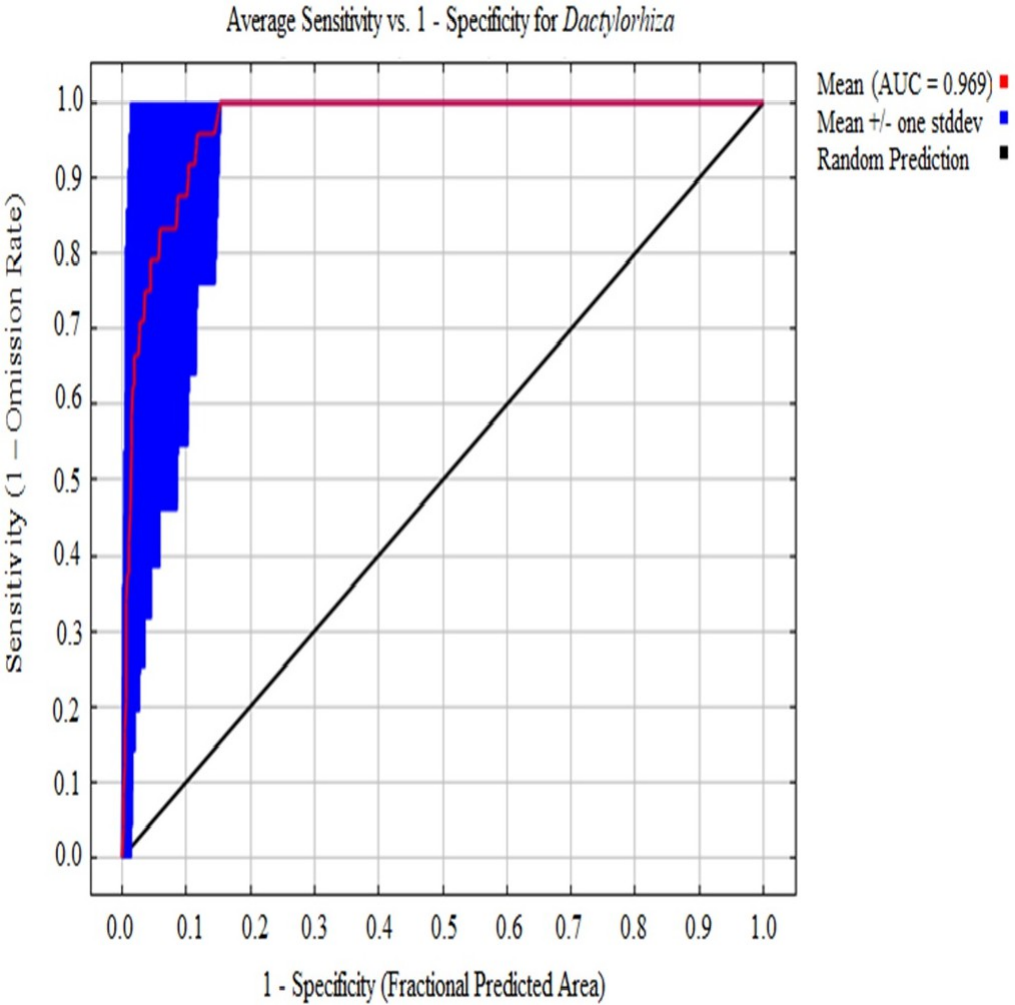
Receiver operating characteristic curve with area under the curve (AUC) signifying model robustness.

The results obtained by an ecological model are judged for their performance based on complex algorithm tests and model validation. The threshold-independent ROC showed that the average AUC yielded satisfactory results of 0.96 (Fig 3), which falls under ‘very good’ (0.95 < AUC < 1.0) model performance based on Thuiller et al. (2005) [42] classification. The confusion matrices for the current prediction model calculated the model’s sensitivity and specificity to be 0.79 and 0.95, respectively. With these matrices in place, the model performance (i.e., TSS) was calculated (sensitivity + specificity—1). The TSS for the current model was computed to be 0.74, which indicates that the model’s overall performance was good, based on Allouche et al. (2006) [45] criteria.

**Fig 4:**
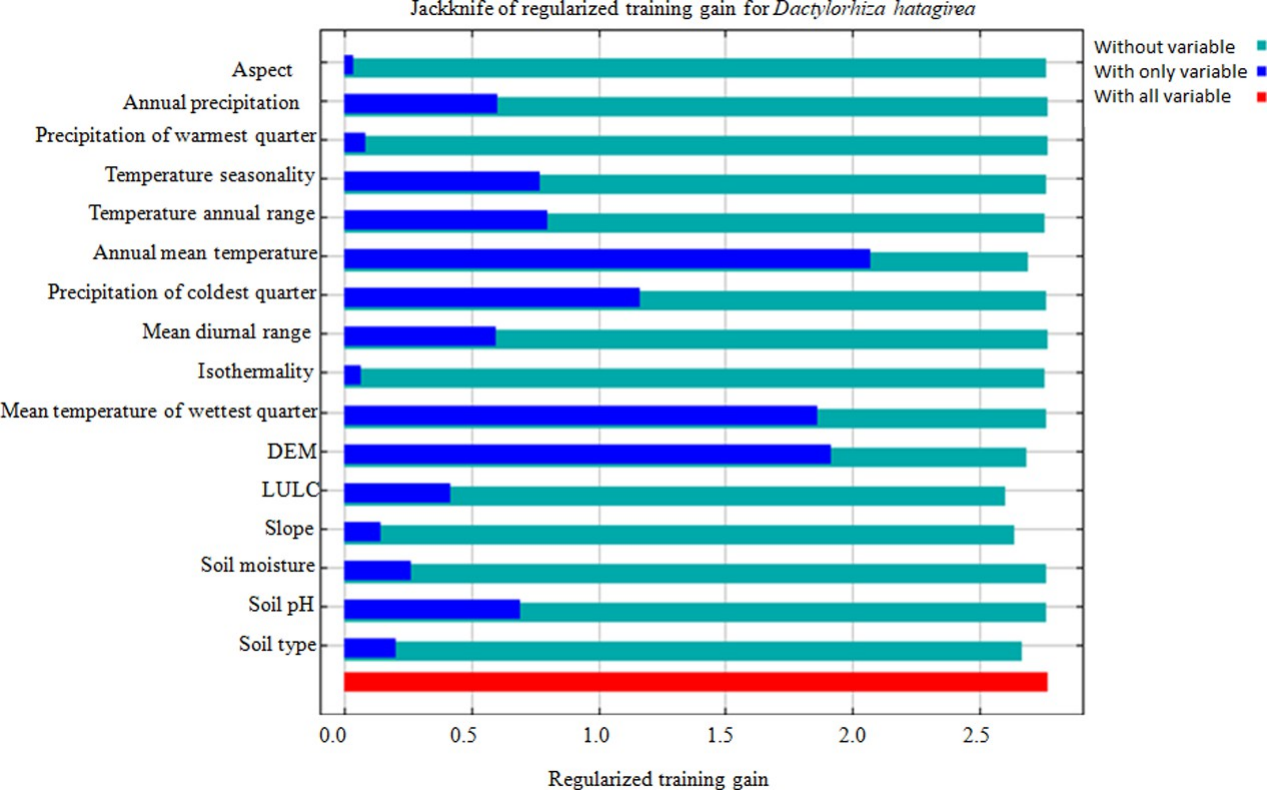
Results of jackknife test highlighting the relative importance of each variable when used in isolation.

**Fig 5:**
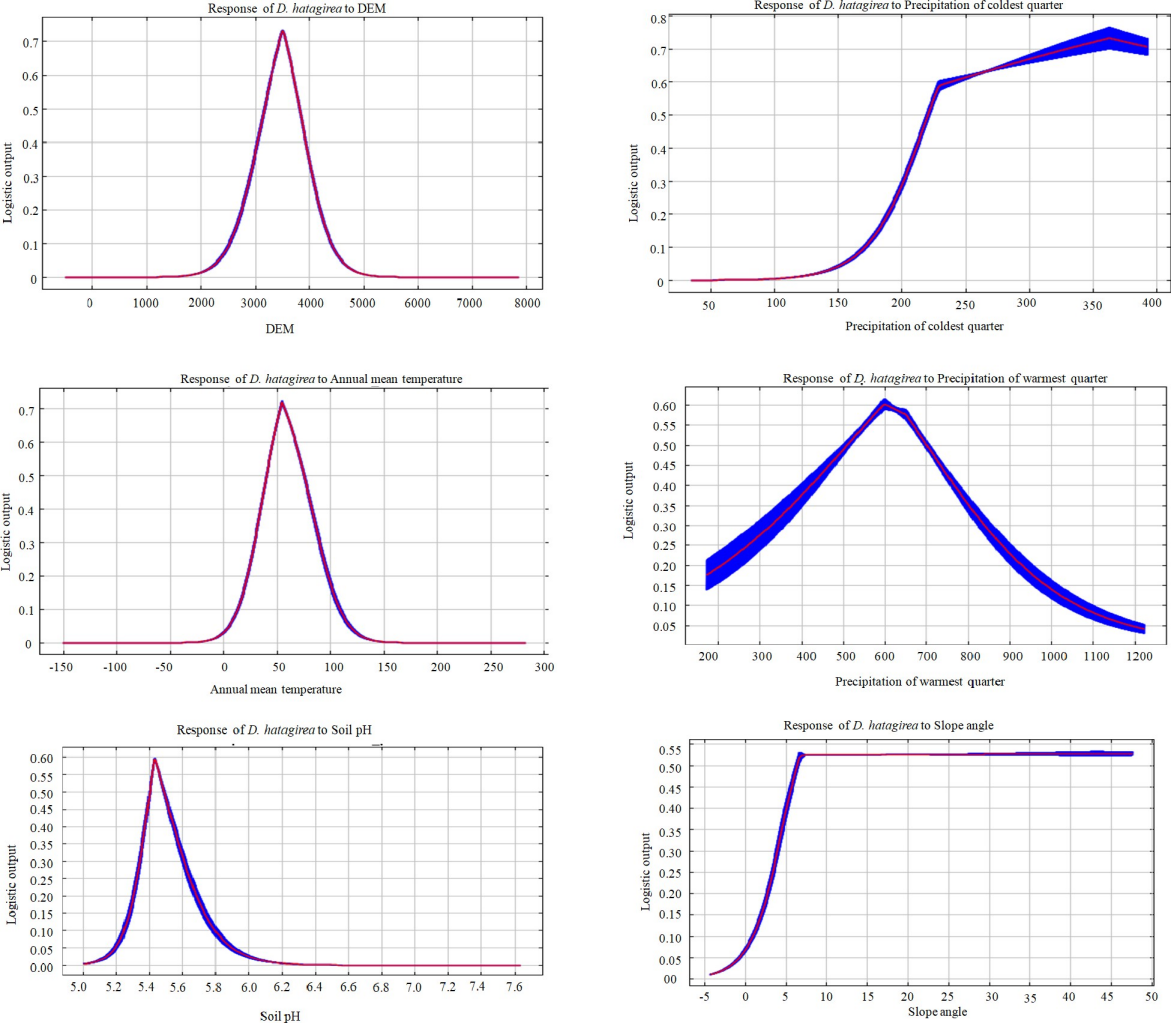
Response curves of six environmental predictors and their relationships with the probability of the target species suitability range.

The variable contributions analysis highlights; elevation had the most (34.85%) influential effect followed by precipitation of coldest quarter (Bio 19; 23.04%), soil type (8.77%), LULC (8.26%), annual mean temperature (Bio 1; 5.51%), temperature seasonality (Bio 4; 5.11%), precipitation of warmest quarter (Bio 18; 4.38%) and Slope (3.06%) (Table 1). The variables mentioned above cumulative contributions stood at ~93% to the modeled potential climatic niche of *D*. *hatagirea*. Similarly, Jackknife analysis indicates annual mean temperature (Bio 1), elevation, mean temperature of the wettest quarter (Bio 8), and precipitation of coldest quarter (Bio 19) as most important predictor variables (Fig 4). These variables provide useful and distinctive information defining the *D*. *hatagirea* distributions when used in isolation. Variables like soil type, LULC, temperature seasonality (Bio 4), and annual temperature range (Bio 7) showed considerable change and showed moderate gain when used separately (Fig 4). Furthermore, the quantitative relationship between the logistic probability and input variables are depicted as response curves (Fig 5). A geomorphic variable, such as elevation, was one of the key variables that describe the present and future spatial distributions of *D*. *hatagirea*. Response curves analysis reveals average altitude ranged from 2800 m to 4500 m, precipitation of coldest quarter (Bio 19) ranged from (150 mm to 380 mm), annual mean temperature (Bio 1) ranged in between (0 – 25°C). Likewise, precipitation of the warmest quarter (Bio 18) ranged in between (200 mm-1100 mm), soil pH (5–6), and slope angle ranged from (5° - 45°). Thus, all the identified variables estimate the important climatic attributes that potentially influence the distribution of *D*. *hatagirea* in northwestern Himalaya, India.

### 3.3. Current predicted potential distribution of climatically suitable areas

**Fig 6:**
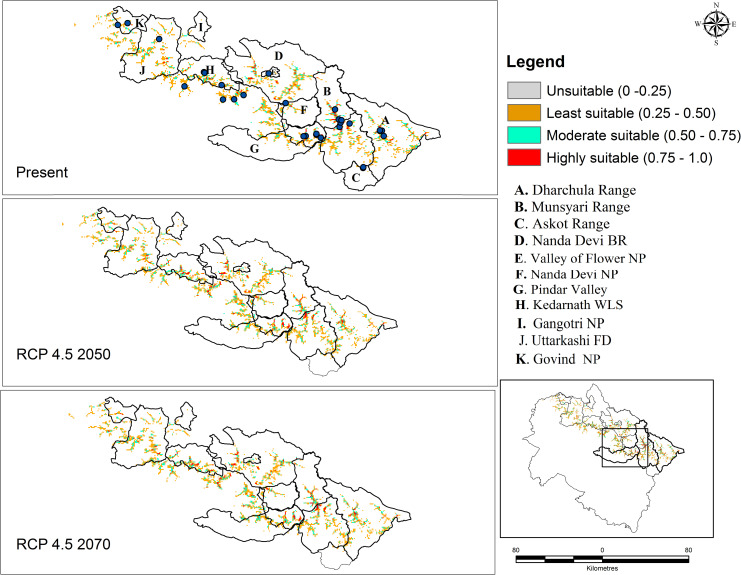
Potential geographic distributions of current and under RCP 4.5 (2050 and 2070) of *D*. *hatagirea* in the Uttarakhand state. Maps in Fig 6 are generated with ArcGIS version 10.3 (ESRI, CA, USA).

Habitat suitability of *D*. *hatagirea* was determined based on threshold interval classification (TIC). Of these, maximum of 96.18% (51637 sq. km) of the geographic region was predicted to be unsuitable (TIC < 0.25), followed by 3.09% (1664 sq. km) with least habitat suitability (0.25 < TIC < 0.50), and moderate suitability being 0.47% (255 sq. km). The potential habitat with high suitability accounts for only 0.24% (131 sq. km) of the state’s total geographic area (Table 2). The areas with high habitats suitability (TIC > 0.75) are mainly concentrated in the forest range like Dharchula and Munsyari range, Pindar valley, Kedarnath Wildlife Sanctuary, West of Nanda Devi Biosphere reserve, and Uttarkashi forest division (Fig 6). Likewise, moderate habitat suitability was located in GovindVihar National Park, Uttarkashi forest division, Kedarnath Wildlife Sanctuary, Nanda Devi Biosphere Reserve, and National Park, Pindar valley and in the forest range of Dharchula and Munsyari range.

**Table 2:** Predicted habitat suitability area (km^2^) of *D*. *hatagirea*, in the present and future climate change scenarios (RCP 4.5–8.5; 2050 and 2070).

		Present	RCP 4.5 2050	RCP 8.5 2050	RCP 4.5 2070	RCP8.52070
Range	Area (km^2^)					
0.00–0.25	Not Suitable	51637	51478	51526	51513	51564
0.25–0.50	Low Suitable	1664	1777	1734	1729	1684
0.50–0.75	Moderately Suitable	255	279	281	285	292
0.75–1.00	Highly Suitable	131	153	146	160	147
Total		53687	53687	53687	53687	53687

### 3.4. Future projection of climatically suitable areas of *D*. *hatagirea* distribution

**Fig 7:**
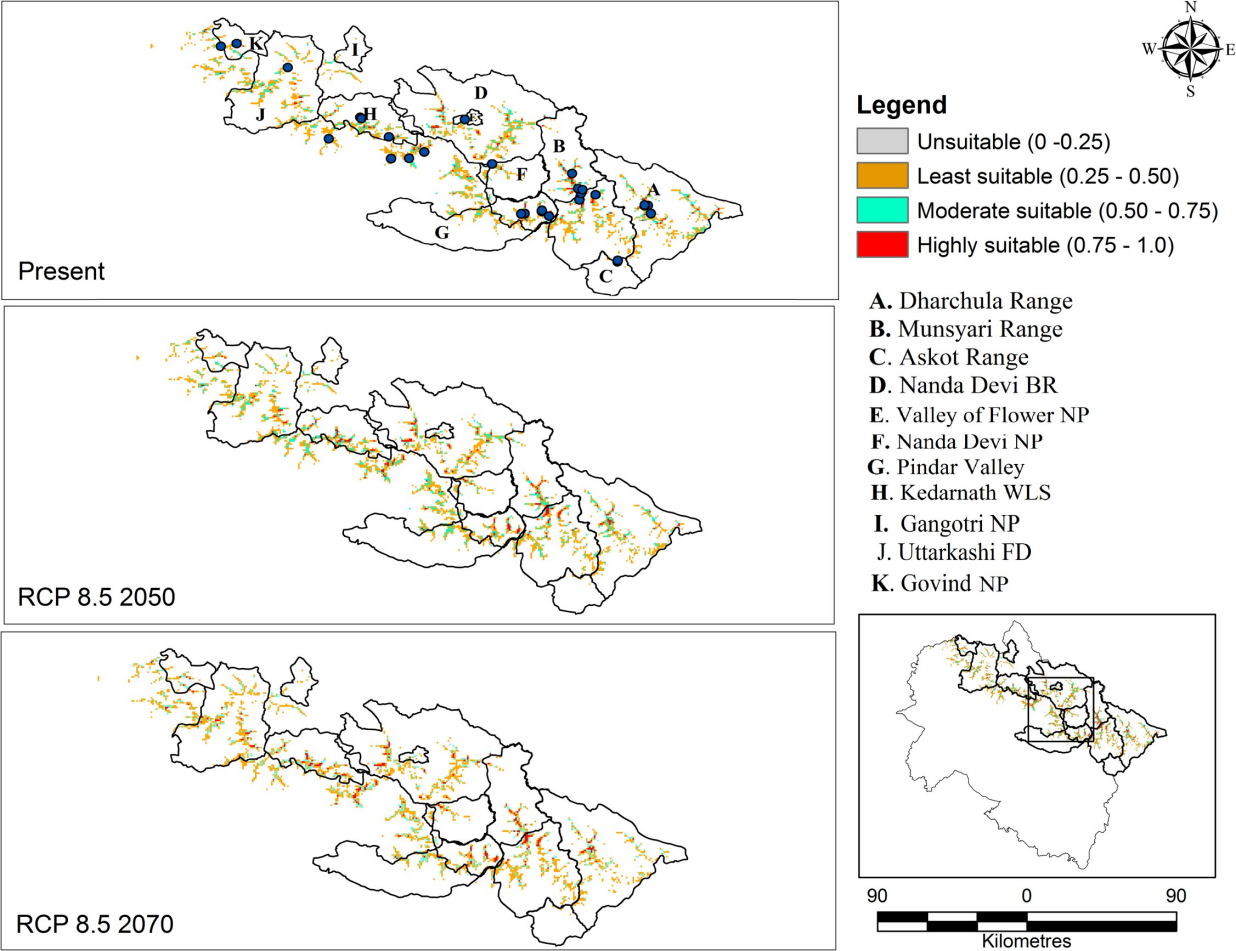
n-depth view of predicted future habitat for *D*. *hatagirea* under RCP 8.5 (2050 & 2070). **I** Maps in Fig 6 are generated with ArcGIS version 10.3 (ESRI, CA, USA).

Future projection habitat suitability map under the CCSM4 model for RCP 4.5 and RCP 8.5 (2050 and 2070) is very similar to the current distribution (Table 2). The present study results depict the geographic distribution of the species would expand under predicted levels of climate change (RCP 4.5 and RCP 8.5) compared with the current potential distribution (Figs 6 and 7). High habitat suitability under the RCP 4.5 scenario; predicts an increase of 0.4% (22.10 sq. km) for 2050 and 0.3% (15 sq. km) for 2070. Under the RCP 8.5 projection, an increase of 0.5% (29 sq. km) is expected in 2050 and 0.27% in 2070. Although, the potential high suitability increases under both the scenarios (RCP 4.5 and 8.5) when compared with the current prediction, the rate of increase for the year 2050 is comparatively higher, after which (towards 2070) it showed a decreasing trend.

### 3.5. Shifts in habitat suitability under the climate change scenarios

The final output maps (current and future scenarios) were employed to find out habitats that will remain stable, gains in habitat area, habitat loss, and unsuitable habitat as part of computing change analysis (in sq. km) (Table 3) (S1 and S2 Figs). The change analysis highlights; only 1992 sq. km of stable habitat (maximum) under RCP 4.5 (2050) then under RCP 8.5 (2070) with a minimum stable habitat of 1928 sq. km. Stable habitats showed a decreasing trend with the climate projection model. Likewise, under RCP 4.5 (2050), an area of 107 sq. km was recorded as a gain in habitat, followed by 102 sq. km in 2050 (RCP 8.5) and 93 sq. km (RCP 8.5) in 2070. Habitat gain indicates the region becomes more suitable for the species under future climatic conditions. Besides these, habitat losses (contraction) were recorded. Of these, maximum (112 sq. km) contraction was seen in 2070 (RCP 4.5), followed by 110 sq. km in 2050 (RCP 4.5) and 105 sq. km in 2050 (RCP 8.5) (Table 3). The contraction in habitats was mainly recorded from the low habitat suitability class. A detailed shift in degrees in a different class is tabulated in Table 4.

**Table 3:** Habitat transformation (km^2^) as predicted by comparison with present distribution using RCP 4.5–8.5 (2050 and 2070).

	RCP 4.5 2050	RCP 4.5 2070	RCP 8.5 2050	RCP 8.5 2070
Gain	107	102	98	93
Loss	110	105	112	103
Stable	1992	1966	1951	1928
Unsuitable	51478	51514	51526	51564
Total area	53687	53687	53687	53687

**Table 4:** Change detection of suitability class by comparison with present distribution as computed in Km^2^ under RCP 4.5 and RCP 8.5.

	RCP 4.5 2050	RCP 4.5 2070	RCP 8.5 2050	RCP 8.5 2070
Suitability class	Shift in the area (km^2^)			
Unsuitable	51478.4	51526	51513.8	51563.9
Unsuitable to low suitable	1099	1086	1079	1065.5
Moderate suitable	142	137	135	136
Low suitable	362	359	360	352.5
New low suitable	106.8	98.1	102	92.5
Contraction	109.7	112	105.3	102.5
Low to unsuitable	75.8	65.5	60.5	57.5
Low to moderate suitable	137	126	131	128
Moderate to low suitable	23.5	13.4	21.5	13.4
Moderate to highly suitable	52.9	54	56.4	50.1
Unsuitable to moderate suitable	0	18	19	27.5
Low to highly suitable	59.2	62	59.6	63.4
Unsuitable to highly suitable	41	30	44	33.5
Total area	53687	53687	53687	53687

## 4. Discussion

The susceptibility to climate change is now reflected in spatial distribution and forest ecosystem vulnerability across the globe [46]. Climate change is projected to be a ‘dominant stressor’ under different climate projection models in the latter half of the 21^st^ century [47]. As there is no denial of the reality of climate change, attention is now being actively paid to formulate some mitigation measures to maintain the stability of an ecosystem. Species distribution models in this direction have contributed immensely by providing reliable information about the potentially suitable habitats of sensitive/ vulnerable species or communities that need priority attention. The present study investigates the habitat suitability of *D*. *hatagirea* under a changing climate scenario using a species distribution model. The study provides a detailed map of the current and future species distribution in light of climate changes (Figs 6 and 7). *Dactylorhiza hatagirea* distribution was mostly explained by topographical variables rather than bioclimatic variables (Table 1). Among the topographical factors, elevation (34.85%) was the most dominant contributing factor, followed by soil type (8.77%) and LULC (8.26%), while among the bioclimatic variables, precipitation of coldest quarter (Bio 19; 23.04%), annual mean temperature (Bio 1; 5.51%), and temperature seasonality (Bio 4; 5.11%) were most prevalent. These physiographic factors, along with topographical features (i.e., elevation, land use characteristics, slope angle, and aspect), and bioclimatic parameters, are reported to have pronounced effects on the pattern of species distribution and community structure in the alpine meadows [35, 48]. Such factors play a key role in the alpine biodiversity where the species are skewed more towards particular habitats (i.e., moist and marshy habitat) than open grasslands or rugged terrains [14, 49].

Previous findings are in line with this study, wherein altitude and bioclimatic variables (temperature and precipitation) were reported to play a major role in the distribution and population structure of *D*. *hatagirea* [50–53]. Among these, Thakur et al. (2021) [52] reported precipitation of the coldest quarter (Bio 19) as the most significant bioclimatic variable that influences *D*. *hatagirea* distribution as obtained in this study. The ecological relevance of Bio 19 in the target species distribution was accredited to its marshy habitat, for which precipitation (in the form of snow) during the winter season could have a direct role in recharging groundwater and maintaining desired soil moisture [52, 54]. Blinova (2008) [55] and Sletvold et al. (2010) [56] have shown that temperature (of the growing season) and precipitation are responsible for the persistence of *Dactylorhiza* populations. Further, a study by Shrestha et al. (2021) [53] from Nepal postulated a different stance where annual mean temperature (Bio1), precipitation seasonality (Bio15), and annual precipitation (Bio12) are the most significant variables in the target species distribution, while, precipitation in the coldest quarter (Bio19), precipitation in the driest quarter (Bio17), and the environmental layer were of intermediate significance. Similar to our findings, Rana et al. (2020) [3], using ensemble species distribution modelling (eSDM), reported elevation (30.97%) as the major dominant contributing variable, while amongst the bioclimatic variables, the mean temperature of the wettest quarter (Bio 8; 24.69%) and annual precipitation (Bio12; 21.11%) were the key contributing variables. Given the understanding from the above and several other studies on terrestrial orchids, other than biotic elements, temperature (of the growing season) and precipitation strongly modulate or affect their distribution. Thus, any significant climate change can be envisaged to have a magnified impact on these species overall distribution and growth performance. An observational study in the laboratory conditions noted maintaining the live germplasm of the species at controlled temperature chambers, i.e., 20°C, 30°C and in glasshouse condition (>35°C), revealed better growth performance and flower development at 20°C only. In comparison, growth ceased after the initial leaf development stage and later perished in temperature above 35°C, suggesting species narrow temperature tolerance regime for growth and perpetuation. Similar findings are reported elsewhere, where an excessive rise in temperatures was reported to affect the vegetative growth flower bud differentiation negatively and preclude regeneration of *Paeonia delavayi* [57], *Camellia sinensis* [37], *Dalbergia cultrata* [58], *Rosa arabica* [59], *Hippophae salicifolia* [60], *Fritillaria cirrhosa* [61], *Rhododendron niveum* [62], and *Ilex khasiana* [43].

In addition to the above, soil type, moisture ratio, and soil pH [49] are the other vital decisive variables that posture or play a role in shaping the distribution of *D*. *hatagirea* (Table 3). In a study by Thakur et al. (2021) [52] revealed that the populations of *D*. *hatagirea* flourished in soils rich in organic matter (i.e., loamy sand to sandy loam) and had adequate soil moisture (~ 63.23%). Also, Shrestha et al. (2021) [53] emphasized geological substrate and soil properties to be the key factors determining the distribution of the species. Similar observation was recorded during our ecological assessment surveys wherein the target species has luxuriant growth in moist sites along the hill slopes, dodged within narrow streams, and on open undulating meadows with high soil moisture [49]. Much of these habitats had slope steepness angles ranging from gentle (14°) to moderate slope (41°) angle, while north/ north-east aspects were most prevalent [49]. These variables were identified as factors of importance in our study, considering their role in posturing the vegetation reserve in the alpine habitats. Therefore, their role cannot be ignored in the Himalayas context, where the influence of the microclimatic conditions edaphic factors largely prevail [63].

In recent years, rapid climate changes in the Himalayas have resulted in distributional changes for a wide range of taxa [64]. The aftermath of these events has seriously impacted the geographical distribution of species, with reports of some species migrating to higher elevations [1, 57, 65–68]. The alpine species in the Himalayas are habitat-specific and have a narrow distributional range; therefore, they are more vulnerable to extinction. The disproportionate effects on these species migrating to high latitude or elevation are attributed to climate change [65]. Our prediction showed that the shift in suitable habitat distribution to high elevations would gradually become more significant using the climate projection model. Model predicted climatically suitable habitat would expand under the RCP 4.5 and RCP 8.5 climate scenario toward north/ northeast direction and would become invariably less suitable at the lower altitudes (Figs 6 and 7). Further, we speculate that the north/ northeastward shift observed in our study could also be attributed to; North-facing aspects in the mountains are associated with higher soil nutrient content, higher biomass, coverage, height, species diversity than south-facing slopes [49, 69, 70]. Climate warming and shifting of species towards north/ northeast in the Himalayas is reported [3, 35, 71, 72]. In Nepal, Shrestha et al. (2021) [53] projected that the target species would be elevated to 5000 m in the future, a substantial change when compared with the present distribution at 4000 m. A similar prediction for the species has been made elsewhere [3, 50].

Likewise, the change analysis, i.e., stable area, habitat loss, and habitat gain area, was carried out for RCP 4.5 and RCP 8.5 (Table 3). The area under stable habitat under RCP 4.5 (2050) was invariably higher RCP 8.5 (2050 and 2070); suggesting changes in climatic parameters and land use characteristics over higher emission rate would negatively impact the suitability of the habitat. Other than these, model prediction advocates climatic changes will also bring new areas (habitat gain) under habitat suitability class, with the highest gain observed under lower emission rate, i.e., RCP 4.5 than RCP 8.5. The study prospects the RCP4.5 scenario to be more favorable for *D*. *hatagirea* than RCP 8.5 in the northwestern part of IHR, thus providing an expansion scope. Meanwhile, warming of lower elevation or areas where the species is currently found explains the climatic niche loss in the future. The result obtained by overlaying the projected layer in the current and future climatic scenarios supports the projection made by Rana et al. (2021) [3] in the Nepal Himalaya using ensemble modelling (eSDM) approaches. Shreshta et al. (2021) [53] proposed a contrasting viewpoint with HadGEM2-ES, CCSM4, and BCC-CSM1-1 simulation model in a different study. Of the three models, BCC-CSM1-1 and CCSM4 simulations anticipated the species to lose 61–85% and 71–98% of its niches by 2070, whereas, with HadGEM2-ES, the species will lose all preferred niches by 2070 in RCP4.5 and RCP8.5 scenarios. In such conditions, parallel studies on community structure, biotic interactions, plant-pollinator or plant-mycorrhizal fungi interactions, population status, and habitat characteristics are also necessary. Hence, an integrated model with both the elements (biotic and abiotic) and their cumulative effects needs further investigation. This is notable because they play an important role in the persistence of *D*. *hatagirea* populations [52].

Along with climate change, irrational harvesting practices of MAPs as a result of increased market demand for herbal medicine and lack of knowledge/ awareness on sustainable harvesting led to serious habitat degradations in the Himalayan region [49]. Similarly, changes in LULC, anthropogenic interference (i.e., habitat degradation and fragmentation, unscientific collection, pre-mature tuber overharvesting, grazing), interspecies competition (i.e., mainly habitat engulfed by *Persicaria wallichii*), and poor seed germination has seriously impacted the relative distribution of *D*. *hatagirea* in the study area [49]. The present study provides an overview of habitat suitability and likely changes with projected climate scenarios in conjunction with changes in the species geomorphologic profile. In this context, the information provided in the present study could be beneficial in various conservation initiatives at the local and regional levels in West Himalaya. The study conducted has some limitations as the model developed to forecast the fundamental niche of the species rather than the realized niche. The species realized niche might not be the same as predicted in our model prediction results. Another limitation is that this study modeled the habitat suitability of *D*. *hatagirea* at different time scales (2050 & 2070) based on abiotic factors but did not consider biotic factors such as plant-mycorrhizal association or plant-pollinator interaction. As a result, potentially suitable area of the species might be overestimated, as biotic interaction, especially mycorrhizal association, play a crucial role in plant establishment in the family Orchidaceae [53, 73].

## 5. Conclusion

*Dactylorhiza hatagirea*, an endemic high-value Himalayan medicinal species, is under great stress and needs urgent attention. The perturbance of climate change has added extra pressure on the species allied with high degrees of anthropogenic stress. Therefore, the present study provided a clear overview of the habitat suitability for *D*. *hatagirea* and predicted the potential impacts of future climate on their distribution in Western Himalaya. The species potential distributions are explained mainly by elevation, precipitation of the coldest quarter, soil moisture, LULC, and mean annual temperature. The study reveals that *D*. *hatagirea* has approximately about 131 sq. km as its fundamental niche with high (>0.75) habitat suitability, which corroborates to about 0.24% of the total geographic area of the state. Under future projections RCP 4.5 and RCP 8.5, species distribution is projected to be very similar to the current distribution, except shifting species in a northward direction to higher elevation is expected. Furthermore, most predicted potential habitats fall within areas with anthropogenic encroachment leading to habitat degradation, unscientific and destructive harvesting practices, grazing, etc. Considering these, it is anticipated that the species may lose much of the habitat due to anthropogenic activities, while climate change impact will invariably be more at comparatively lower altitudes. Thus, it is imperative to undertake appropriate conservation steps and interventions like reintroduction/ augmentation programs. Besides these, the dependent communities awareness and sensitization curriculum holds utmost importance towards sustainable utilization. The model generated suitability maps can be of significant help to various policy prescribing National agencies, i.e., National Medicinal Plant Board (NMPB), Ministry of Environment Forest and Climate Change (MOEF &CC), Department of Science & Technology (DST). At the State level, the State Government has control over Forest Department and NGOs, thus ensuring and safeguarding the overall health of our forests and meadows.

## Supporting information

S1 Table Multi-collinearity test by using cross-correlations (Pearson correlation coefficients, r) among environmental variables using ENM Tools. (DOCX)

S1 Fig Habitat transformations with respect to present distribution into different classes as depicted under RCP 4.5 (2050 and 2070). Maps in S1 Fig are generated with ArcGIS version 10.3 (ESRI, CA, USA).
(TIF)

S2 Fig A comparative studies on habitat transformations between present distribution and under RCP 8.5 (2050 and 2070). Maps in S2 Fig are generated with ArcGIS version 10.3 (ESRI, CA, USA).
(TIF)

## References

[1] Lenoir J, Gégout JC, Marquet P, De Ruffray P, Brisse HJS. A significant upward shift in plant species optimum elevation during the 20th century. Science. 2008; 320: 1768–1771. 10.1126/science.1156831 18583610

[2] You JL, Qin XP, Ranjitkar S, Lougheed SC, Wang MC, Zhou W, et al. Response to climate change of montane herbaceous plants in the genus Rhodiola predicted by ecological niche modelling. Sci. Rep. 2018; 8: 5879. 10.1038/s41598-018-24360-9 29651147 PMC5897335

[3] Rana SK, Rana HK, Ranjitkar S, Ghimire SK, Gurmachhan CM, O’Neill AR, et al. Climate-change threats to distribution, habitats, sustainability and conservation of highly traded medicinal and aromatic plants in Nepal. Ecol. Indic. 2020; 115: 106435. 10.1016/j.ecolind.2020.106435.

[4] Carrasco J, Price V, Tulloch V, Mills M. Selecting priority areas for the conservation of endemic trees species and their ecosystems in Madagascar considering both conservation value and vulnerability to human pressure. Biodivers. Conserv. 2020; 29(6): 1841–1854. 10.1007/s10531-020-01947-1.

[5] Hamid M, Khuroo AA, Charles B, Ahmad R, Singh CP, Aravind NA. Impact of climate change on the distribution range and niche dynamics of Himalayan birch, a typical treeline species in Himalayas. Biodivers. Conserv. 2019; 28(8): 2345–2370. 10.1007/s10531-018-1641-8.

[6] Singh L, Tariq M, Sekar KC, Bhatt ID, Nandi SK. Ecological niche modelling: an important tool for predicting suitable habitat and conservation of the Himalayan medicinal herbs. ENVIS Bull. Himal. Ecol. 2017; 25, 154–155.

[7] Brandt JS, Haynes MA, Kuemmerle T, Waller DM, Radeloff VC. Regime shift on the roof of the world: Alpine meadows converting to shrublands in the southern Himalayas. Biol. Conserv. 2013; 158:116–27. 10.1016/j.biocon.2012.07.026.

[8] Negi VS, Tiwari DC, Singh L, Thakur S, Bhatt ID. Review and synthesis of climate change studies in the Himalayan region. Environ. Dev. Sustain. 2021; 1–32. 10.1007/s10668-021-01880-5.

[9] Shrestha UB, Gautam S, Bawa KS. Widespread climate change in the Himalayas and associated changes in local ecosystems. PLoS One. 2012; 7(5):e36741. 10.1371/journal.pone.0036741 22615804 PMC3352921

[10] Kumar KR, Sahai AK, Kumar KK, Patwardhan SK, Mishra PK, Revadekar JV, et al. High-resolution climate change scenarios for India for the 21st century. Curr. Sci. 2006; 334–45. https://www.jstor.org/stable/24091867.

[11] Pandita S, Kumar V, Dutt HC. Environmental variables vis-a-vis distribution of herbaceous tracheophytes on northern sub-slopes in Western Himalayan ecotone. Ecol. Process. 2019; 8(1):1–9. 10.1186/s13717-019-0200-x.

[12] Samant SS, Dhar U, Palni LMS. Medicinal plants of Himalaya: diversity, distribution potential values. Gyanodaya Prakashan; 1998.

[13] Rodgers WA, Panwar HS. Planning a wild life protected area network in India. The report, Wild Life Institute of India, Dehradun; 1998.

[14] Kala CP. Indigenous uses, population density, and conservation of threatened medicinal plants in protected areas of the Indian Himalayas. Conserv. Biol. 2005; 19(2): 368–378. 10.1111/j.1523-1739.2005.00602.x.

[15] Mehta P, Sekar KC, Bhatt D, Tewari A, Bisht K, Upadhyay S, et al. Conservation and prioritization of threatened plants in Indian Himalayan Region. Biodivers. Conserv. 2020; 29(6): 1723–1745. 10.1007/s10531-020-01959-x.

[16] Guisan A, Thomas C, Edwards JTC, Hastie T. Generalized linear and generalized additive models in studies of species distributions: setting the scene. Ecol. Modell. 2002; 157(2–3): 89–100. 10.1016/S0304-3800(02)00204-1.

[17] Booth TH. Estimating potential range and hence climatic adaptability in selected tree species. Forest Ecol. Manage. 2016; 366, 175–183. doi: 10.1016/j.foreco.2016.02.009

[18] Guisan A, Zimmermann NE. Predictive habitat distribution models in ecology. Ecol. Modell. 2000; 135(2–3): 147–186. 10.1016/S0304-3800(00)00354-9.

[19] Pearson RG, Raxworthy CJ, Nakamura M, Peterson AT. Predicting species distributions from small numbers of occurrence records: a test case using cryptic geckos in Madagascar. J. Biogeogr. 2007; 34:102–117. 10.1111/j.1365-2699.2006.01594.x.

[20] D’Elia J, Haig SM, Johnson M, Marcot BG, Young R. Activity-specific ecological niche models for planning reintroductions of California condors (*Gymnogyps californianus*). Biol. Conserv. 2015; 184: 90–99. 10.1016/j.biocon.2015.01.002.

[21] Volis S. Conservation-oriented restoration–how to make it a success? Isr. J. Plant Sci. 2016; 63(4): 276–296. 10.1080/07929978.2016.1255020.

[22] CAMP. Conservation assessment and management prioritization for the medicinal plants of Jammu & Kashmir, Himachal Pradesh & Uttaranchal. In: Ved D. K., Kinhal G. A., Ravikumar K., Prabhakaran V., Ghate U., Shankar R. V., Indresha J. H., (edition), FRLHT, Bangalore. India; 2003.

[23] Dhiman N, Sharma NK, Thapa P, Sharma I, Swarnkar MK, Chawla A, et al. *De novo* transcriptome provides insights into the growth behavior and resveratrol and trans-stilbenes biosynthesis in *Dactylorhiza hatagirea*- An endangered alpine terrestrial orchid of western Himalaya. Sci. Rep. 2019; 9: 13133. 10.1038/s41598-019-49446-w 31511556 PMC6739469

[24] Goraya GS, Ved DK. Medicinal plants in India: an assessment of their demand and supply. National medicinal plants board, Ministry of Ayush, Government of India, New Delhi and Indian Council of Forestry Research and Education, Dehradun; 2017.

[25] FSI. India State of Forest Report. URL http://fsi.nic.in/http://fsi.nic.in/forest-report-2019.

[26] Malik A, Kumar A, Guhathakurta P, Kisi O. Spatial-temporal trend analysis of seasonal and annual rainfall (1966–2015) using innovative trend analysis method with significance test. Arab. J. Geosci. 2019; 12: 328. 10.1007/s12517-019-4454-5.

[27] http://www.imdpune.gov.in/hydrology/rainfall%20variability%20page/rainfall%20trend.html (accessed on 20th April 2020).

[28] Rawat GS. (2005) Alpine Meadows of Uttaranchal. Bishen Singh Mahendra Pal Singh, Dehradun; 2005.

[29] Phillips SJ, Anderson RP, Schapire RE. Maximum entropy modeling of species geographic distributions. Ecol. Modell. 2006; 190(3–4): 231–259. 10.1016/j.ecolmodel.2005.03.026.

[30] https://www.gbif.org/ (accessed on 03 March, 2020).

[31] http://www.worldclim.org/ (accessed on 16 May, 2020).

[32] https://soilgrids.org/ (accessed on 21 May, 2020).

[33] http://www.landcover.org/ (accessed on 22 May, 2020).

[34] http://srtm.csi.cgiar.org (accessed on 16 May, 2020).

[35] Chitale VS, Behera MD, Roy PS. Future of endemic flora of biodiversity hotspots in India. PLoS One. 2014; 9(12): e115264. doi: 10.1371/journal.pone.0115264 25501852 PMC4264876

[36] IPCC. IPCC’s Fifth Assessment Report (AR5), Intergovernmental Panel on Climate Change. Geneva, Switzerland; 2014.

[37] Jayasinghe SL, Kumar L. Modeling the climate suitability of tea [*Camellia sinensis* (L.) O. Kuntze] in Sri Lanka in response to current and future climate change scenarios. Agric. For Meteorol. 2019; 272–273: 102–117. 10.1016/j.agrformet.2019.03.025.

[38] http://www.cs.princeton.edu/wschapire/Maxent/ (accessed on 05 June, 2020).

[39] Guisan A, Thuiller W. Predicting species distribution: offering more than simple habitat models. Ecol. Lett. 2005; 8(9): 993–1009. 10.1111/j.1461-0248.2005.00792.x 34517687

[40] Elith J, Graham CH, Anderson RP, Dudik M, Ferrier S, Guisan A, et al. Novel methods improve prediction of species’ distributions from occurrence data. Ecography. 2006; 29(2): 129–151. 10.1111/j.2006.0906-7590.04596.x.

[41] Cohen J. A coefficient of agreement of nominal scales. Educ. Psychol. Meas. 1960; 20: 37–46. https://doi.org/10.1177%2F001316446002000104.

[42] Thuiller W, Richardson DM, Pysek P, Midgley GF, Hughes GO, Rouget M. Niche-based modelling as a tool for predicting the risk of alien plant invasions at a global scale. Glob. Chang. Biol. 2005; 11(12): 2234–2250. 10.1111/j.1365-2486.2005.001018.x 34991288

[43] Adhikari D, Barik SK, Upadhaya K. Habitat distribution modelling for reintroduction of *Ilex khasiana* Purk a critically endangered tree species of northeastern India. Ecol. Eng. 2012; 40: 37–43. 10.1016/j.ecoleng.2011.12.004.

[44] Lobo JM, Jiménez-valverde A, Real R. AUC: a misleading measure of the performance of predictive distribution models. Glob. Ecol. Biogeogr. 2008; 17(2): 145–151. 10.1111/j.1466-8238.2007.00358.x.

[45] Allouche O, Tsoar A, Kadmon R. Assessing the accuracy of species distribution models: prevalence, kappa and the true skill statistic (TSS). J. Appl. Ecol. 2006; 43(6):1223–32. 10.1111/j.1365-2664.2006.01214.x.

[46] Pramanik M, Paudel U, Mondal B, Chakraborti S, Deb P. Predicting climate change impacts on the distribution of the threatened Garcinia indica in the Western Ghats, India. Clim. Risk Manag. 2018; 19: 94–105. 10.1016/j.crm.2017.11.002.

[47] Pachauri RK, Allen MR, Barros VR, Broome J, Cramer W, et al. Climate change 2014: synthesis report. Contribution of working groups I, II and III to the fifth assessment report of the Intergovernmental Panel on Climate Change. IPCC. 2014; pp-151.

[48] Schickhoff U, Bobrowski M, Böhner J, Bürzle B, Chaudhary RP, Gerlitz L, et al. Do Himalayan treelines respond to recent climate change? An evaluation of sensitivity indicators. Earth Syst. Dyn. 2014; 5(2): 1407–1461. doi: 10.5194/esd-6-245-2015

[49] Singh L, Bhatt ID, Negi VS, Nandi SK, Rawal RS, Bisht AK. Population status, threats, and conservation options of the orchid *Dactylorhiza hatagirea* in Indian Western Himalaya. Reg. Environ. Chang. 2021; 21:40. doi: 10.1007/s10113-021-01762-6

[50] Kunwar RM, Rimal B, Sharma HP, Poudel RC, Pyakurel D, Tiwari A, et al. Distribution and habitat modeling of *Dactylorhiza hatagirea* (D. Don) Soo, *Paris polyphylla* Sm. and *Taxus* species in Nepal Himalaya, J. Applied Res. Med. Arom. Plants. 2020; 20: 100274. 10.1016/j.jarmap.2020.100274.

[51] Wani IA, Verma S, Mushtaq S, Alsahli AA, Alyemeni MN, et al. Ecological analysis and environmental niche modelling of *Dactylorhiza hatagirea* (D. Don) Soo: A conservation approach for critically endangered medicinal orchid. Saudi J. Biol. Sci. 2021; 28(4):2109–22. doi: 10.1016/j.sjbs.2021.01.054 33911927 PMC8071888

[52] Thakur D, Rathore N, Sharma MK, Parkash O, Chawla A. Identification of ecological factors affecting the occurrence and abundance of *Dactylorhiza hatagirea* (D. Don) Soo in the Himalaya. J. App. Res. Med. Aromat. Plants. 2021; 20:100286. 10.1016/j.jarmap.2020.100286.

[53] Shrestha B, Tsiftsis S, Chapagain DJ, Khadka C, Bhattarai P, et al. Suitability of habitats in Nepal for *Dactylorhiza hatagirea* now and under predicted future changes in climate. Plants. 2021; 10(3): 467. 10.3390/plants10030467 33801220 PMC8000360

[54] Gray DM, Toth B, Zhao L, Pomeroy JW, Granger RJ. Estimating areal snowmelt infiltration into frozen soils. Hydrol. Process. 2001; 15: 3095–3111. 10.1002/hyp.320.

[55] Blinova IV. Populations of orchids at the northern limit of their distribution (*Murmansk Oblast*): Effect of climate. Russ. J. Ecol. 2008; 39(1): 26–33. doi: 10.1007/s11184-008-1005-9

[56] Sletvold N, Grindeland JM, Ågren J. Pollinator-mediated selection on floral display, spur length and flowering phenology in the deceptive orchid *Dactylorhiza lapponica*. New Phytol. 2010; 188(2): 385–392. 10.1111/j.1469-8137.2010.03296.x 20497348

[57] Zhang K, Yao L, Meng J, Tao J. Maxent modeling for predicting the potential geographical distribution of two peony species under climate change. Sci. Total Environ. 2018; 634: 1326–1334. 10.1016/j.scitotenv.2018.04.112 29710632

[58] Liu Y, Huang P, Lin F, Yang W, Gaisberger H, Christopher K, et al. MaxEnt modelling for predicting the potential distribution of a near threatened rosewood species (*Dalbergia cultrata* Graham ex Benth). Ecol. Eng. 2019; 141: 105612. 10.1016/j.ecoleng.2019.105612.

[59] Abdelaal M, Fois M, Fenu G, Bacchetta G. Using MaxEnt modeling to predict the potential distribution of the endemic plant *Rosa arabica* Crép. in Egypt. Ecol. Inform. 2019; 50: 68–75. 10.1016/j.ecoinf.2019.01.003.

[60] Dhyani S, Kadaverugu R, Dhyani D, Verma P, Pujari P. Predicting impacts of climate variability on habitats of *Hippophae salicifolia* (D. Don) (Sea buckthorn) in Central Himalayas: Future challenges. Ecol. Inform. 2018; 48: 135–146. 10.1016/j.ecoinf.2018.09.003.

[61] Zhao Q, Li R, Gao Y, Yao Q, Guo X, Wang W. Modeling impacts of climate change on the geographic distribution of medicinal plant *Fritillaria cirrhosa* D. Don. Plant Biosyst. 2018; 152(3), 349–355. 10.1080/11263504.2017.1289273.

[62] Chhetri B, Badola HK. Predicting suitable habitats for the vulnerable species, *Rhododendron niveum* Hook. f. (Ericaceae) in eastern Himalaya. NeBio. 2017; 8(3): 139–146.

[63] Parajuli R, O’Brien MJ, Timilsina B, Pugnaire FI, Schöb C, et al. Facilitation by a dwarf shrub enhances plant diversity of human-valued species at high elevations in the Himalayas of Nepal. Basic Appl. Ecol. 2021; 54:23–36. 10.1016/j.baae.2021.04.004.

[64] Chen IC, Hill JK, Ohlemuller R, Roy DB, Thomas CD. Rapid range shifts of species associated with high levels of climate warming. Science. 2011; 333, 1024–1026. 10.1126/science.1206432 21852500

[65] Bertrand R, Lenoir J, Piedallu C, Riofrío-Dillon G, De Ruffray P, Vidal C, et al. Changes in plant community composition lag behind climate warming in lowland forests. Nature. 2011; 479: 517–520. 10.1038/nature10548 22012261

[66] Fordham DA, ResitAkçakaya H, Araújo MB, Elith J, Keith DA, Pearson R, et al. Plant extinction risk under climate change: are forecast range shifts alone a good indicator of species vulnerability to global warming? Glob. Chang. Biol. 2012; 18 (4): 1357–1371. 10.1111/j.1365-2486.2011.02614.x.

[67] Telwala Y, Brook BW, Pandit MK. Climate-induced elevational range shifts and increase in plant species richness in a Himalayan biodiversity epicentre. PLoS One. 2013; 8 (2): e57103. 10.1371/journal.pone.0057103 23437322 PMC3577782

[68] Trindade WCF, Santos MH, Artoni RF. Climate change shifts the distribution of vegetation types in South Brazilian hotspots. Reg. Environ. Chang. 2020; 20: 90. 10.1007/s10113-020-01686-7.

[69] Kutiel P, Lavee H. Effect of slope aspect on soil and vegetation properties along an aridity transect. Isr. J. Plant Sci. 1999; 47: 169. 10.1080/07929978.1999.10676770

[70] Paudel S, Vetaas OR. Effects of topography and land use on woody plant species composition and beta diversity in an arid Trans-Himalayan landscape, Nepal. J. Mount. Sci. 2014; 11: 1112–1122. 10.1007/s11629-013-2858-3.

[71] Boisvert-Marsh L, Périé C, de Blois S. Shifting with climate? Evidence for recent changes in tree species distribution at high latitudes. Ecosphere. 2014; 5: 1–33. 10.1890/ES14-00111.1.

[72] Yu F, Wang T, Groen TA, Skidmore AK, Yang X, Ma K, et al. Climate and land use changes will degrade the distribution of *Rhododendrons* in China. Sci. Total Environ. 2019; 659: 515–528. 10.1016/j.scitotenv.2018.12.223 31096381

[73] Tsiftsis S, Djordjević V. Modelling sexually deceptive orchid species distributions under future climates: The importance of plant–pollinator interactions. Sci. Rep. 2020; 10(1):1–2. 10.1038/s41598-020-67491-8 32606363 PMC7327032

