# Supplementary material for: Predicting the potential distribution of Dactylorhiza hatagirea (D. Don) Soo-an important medicinal orchid in the West Himalaya, under multiple climate change scenarios
Source: PLoS One. 2022 Jun 17;17(6):e0269673. doi: 10.1371/journal.pone.0269673 (PMC9205508; doi:10.1371/journal.pone.0269673)
Supplement: S1 Table — (DOCX) [file pone.0269673.s001.docx]

**Table S 1:** Multi-collinearity test by using cross-correlations (Pearson correlation coefficients, r) among environmental variables using ENM Tools

| Variables | BIO1 | BIO2 | BIO3 | BIO5 | BIO6 | BIO7 | BIO12 | BIO8 | BIO9 | BIO10 | BIO11 | BIO18 | BIO13 | BIO14 | BIO15 | BIO16 | BIO17 | SLPE | BIO19 | BIO4 | ELEV |
| --- | --- | --- | --- | --- | --- | --- | --- | --- | --- | --- | --- | --- | --- | --- | --- | --- | --- | --- | --- | --- | --- |
| BIO1 | 1 |  |  |  |  |  |  |  |  |  |  |  |  |  |  |  |  |  |  |  |  |
| BIO2 | 0.360 | 1 |  |  |  |  |  |  |  |  |  |  |  |  |  |  |  |  |  |  |  |
| BIO3 | 0.477 | 0.547 | 1 |  |  |  |  |  |  |  |  |  |  |  |  |  |  |  |  |  |  |
| BIO5 | 0.989 | 0.482 | 0.489 | 1 |  |  |  |  |  |  |  |  |  |  |  |  |  |  |  |  |  |
| BIO6 | 0.989 | 0.237 | 0.472 | 0.958 | 1 |  |  |  |  |  |  |  |  |  |  |  |  |  |  |  |  |
| BIO7 | 0.151 | 0.900 | 0.134 | 0.291 | 0.008 | 1 |  |  |  |  |  |  |  |  |  |  |  |  |  |  |  |
| BIO12 | 0.768 | -0.033 | 0.325 | 0.711 | 0.812 | -0.231 | 1 |  |  |  |  |  |  |  |  |  |  |  |  |  |  |
| BIO8 | 0.996 | 0.425 | 0.483 | 0.996 | 0.975 | 0.226 | 0.737 | 1 |  |  |  |  |  |  |  |  |  |  |  |  |  |
| BIO9 | 0.964 | 0.436 | 0.391 | 0.973 | 0.932 | 0.287 | 0.676 | 0.972 | 1 |  |  |  |  |  |  |  |  |  |  |  |  |
| BIO10 | 0.995 | 0.429 | 0.462 | 0.997 | 0.971 | 0.241 | 0.731 | 0.999 | 0.976 | 1 |  |  |  |  |  |  |  |  |  |  |  |
| BIO11 | 0.997 | 0.309 | 0.486 | 0.977 | 0.997 | 0.086 | 0.793 | 0.988 | 0.950 | 0.986 | 1 |  |  |  |  |  |  |  |  |  |  |
| BIO18 | 0.636 | 0.022 | 0.451 | 0.586 | 0.680 | -0.226 | 0.931 | 0.606 | 0.526 | 0.596 | 0.664 | 1 |  |  |  |  |  |  |  |  |  |
| BIO13 | 0.811 | 0.211 | 0.369 | 0.791 | 0.816 | 0.038 | 0.943 | 0.794 | 0.754 | 0.796 | 0.820 | 0.876 | 1 |  |  |  |  |  |  |  |  |
| BIO14 | -0.468 | -0.663 | -0.533 | -0.532 | -0.407 | -0.503 | -0.175 | -0.486 | -0.455 | -0.491 | -0.451 | -0.199 | -0.423 | 1 |  |  |  |  |  |  |  |
| BIO15 | 0.804 | 0.584 | 0.446 | 0.844 | 0.751 | 0.443 | 0.655 | 0.816 | 0.819 | 0.826 | 0.788 | 0.599 | 0.847 | -0.735 | 1 |  |  |  |  |  |  |
| BIO16 | -0.091 | -0.830 | -0.340 | -0.209 | 0.022 | -0.810 | 0.305 | -0.149 | -0.194 | -0.156 | -0.044 | 0.224 | 0.028 | 0.748 | -0.444 | 1 |  |  |  |  |  |
| BIO17 | -0.091 | -0.830 | -0.340 | -0.209 | 0.022 | -0.810 | 0.305 | -0.149 | -0.194 | -0.156 | -0.044 | 0.224 | 0.028 | 0.748 | -0.444 | ~1 | 1 |  |  |  |  |
| SLPE | -0.582 | -0.464 | -0.258 | -0.622 | -0.530 | -0.402 | -0.331 | -0.600 | -0.602 | -0.607 | -0.562 | -0.252 | -0.472 | 0.545 | -0.628 | 0.377 | 0.377 | 1 |  |  |  |
| BIO19 | -0.294 | -0.783 | -0.413 | -0.393 | -0.199 | -0.714 | 0.012 | -0.334 | -0.336 | -0.342 | -0.260 | -0.033 | -0.279 | 0.923 | -0.674 | 0.903 | 0.903 | 0.495 | 1 |  |  |
| BIO4 | -0.421 | 0.523 | -0.304 | -0.296 | -0.545 | 0.792 | -0.661 | -0.349 | -0.252 | -0.333 | -0.484 | -0.630 | -0.473 | -0.031 | -0.127 | -0.591 | -0.591 | -0.006 | -0.341 | 1 |  |
| ELEV | -0.984 | -0.351 | -0.466 | -0.974 | -0.974 | -0.147 | -0.762 | -0.979 | -0.947 | -0.979 | -0.983 | -0.632 | -0.811 | 0.493 | -0.807 | 0.097 | 0.097 | 0.593 | 0.313 | 0.428 | 1 |
