# Supplementary figures and images for: Predicting the potential distribution of Dactylorhiza hatagirea (D. Don) Soo-an important medicinal orchid in the West Himalaya, under multiple climate change scenarios
Source: PLoS One. 2022 Jun 17;17(6):e0269673. doi: 10.1371/journal.pone.0269673 (PMC9205508; doi:10.1371/journal.pone.0269673)

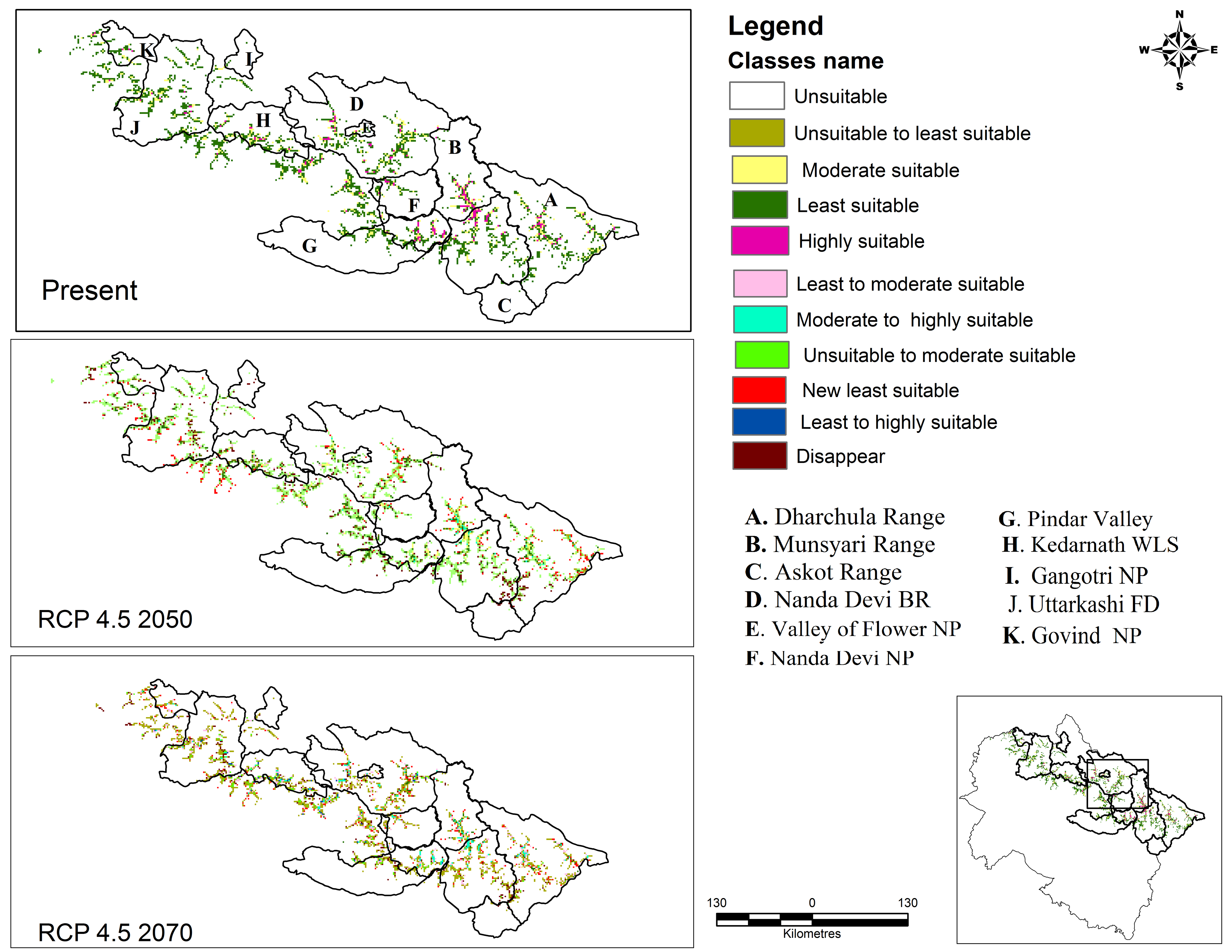

Supplement: S1 Fig — Maps in S1 Fig are generated with ArcGIS version 10.3 (ESRI, CA, USA). (TIF) [file pone.0269673.s002.tif]

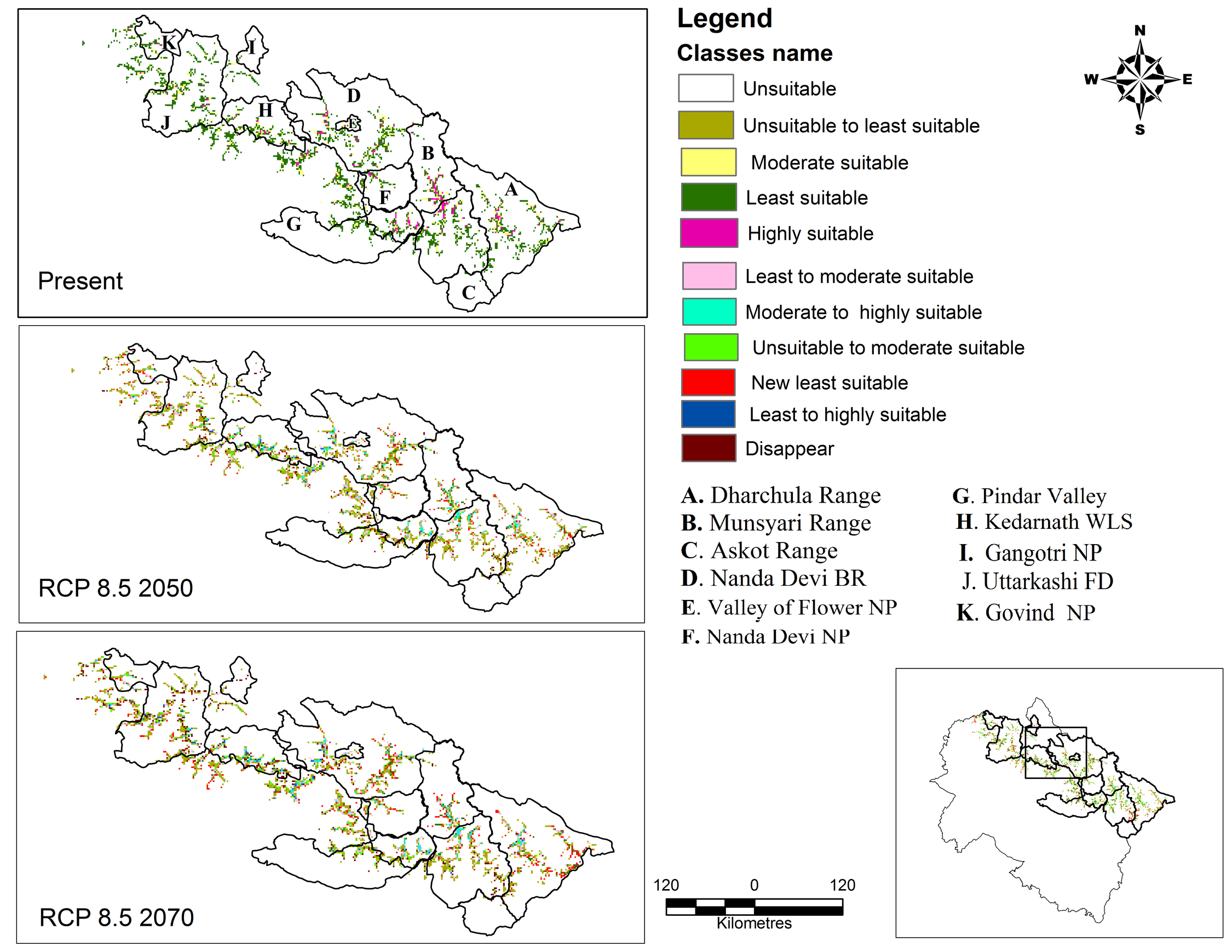

Supplement: S2 Fig — Maps in S2 Fig are generated with ArcGIS version 10.3 (ESRI, CA, USA). (TIF) [file pone.0269673.s003.tif]
