# Supplementary material for: Predicting the potential distribution of Dactylorhiza hatagirea (D. Don) Soo-an important medicinal orchid in the West Himalaya, under multiple climate change scenarios
Source: PLoS One. 2022 Jun 17;17(6):e0269673. doi: 10.1371/journal.pone.0269673 (PMC9205508; doi:10.1371/journal.pone.0269673)
Supplement: Attachment — Submitted filename: Response to Reviewer.docx [file pone.0269673.s004.docx]

**Editorial Desk**

| **S. No** | **Comments/suggestions/ queries** | **Response** |
| --- | --- | --- |
| **1** | PLOS requires an ORCID iD for the corresponding author in Editorial Manager on papers submitted after December 6th, 2016. | ORCID iD of the corresponding author is added |
| **2** | We note that Figures 1, 5, 6, 7, and 8 in your submission contain map images which may be copyrighted. All PLOS content is published under the Creative Commons Attribution License (CC BY 4.0), which means that the manuscript, images, and Supporting Information files will be freely available online, and any third party is permitted to access, download, copy, distribute, and use these materials in any way, even commercially, with proper attribution. | In this regards a separate letter is provided from our end, where we categorically stated that these figures are original and prepared using ArcGis ver. 10.3 software. None of the figures are taken or reproduced from any secondary data source. |

**Reviewer # 1**

| **S. No.** | **Comments/suggestions/ queries** | **Response** |
| --- | --- | --- |
| 1 | Although the authors use a standard methodology, and the models seem to be well constructed, the study has a too narrow scope. It is hard to understand why the authors have limited the niche models to just a very small range of this species (Uttarakhand), as it is actually distributed not only to the Indian Himalayas (as the authors state in lines 91-92), but on a large strip from Pakistan to Mongolia and NE China | As indicated by the reviewer, some insights on the potential distribution of the target species across the Himalayan region was given by Thakur et al. (2021), however, detailed investigation at the regional level was identified as gap area of research.  Resource limitation to validate the model in other part of the Himalayan region, asserts the present study to the state of Uttarakhand. Further, on the realistic side, we have extensively surveyed the state of Uttarakhand for population estimation along the altitudinal gradient and in different habitat types [Singh et al. (2021); doi doi.org/10.1007/s10113-021-01762-6] and validated the model output with the field data.  Needful revision on the manuscript, on the distribution of the target species is incorporated and revised. |
|  | Another large problem is that a very similar paper has been just published | We believe that every study conducted has some merits and limitations. As indicated, previously two papers are published.  In a study by Thakur et al. (2021), emphasized on the ecological factors affecting the occurrence of the species and ground truth for Himachal Pradesh (India) only, although they have model the presence of the species for the entire Himalayan region.  In another study by Chandra et al. (2021), their work was concentrated specifically in the Uttarakhand state and ground truth. Likely impact of climate change on the species suitable habitat and transformation thereafter was the gap area of research.  Thus, taking the learning’s from both the study, the present study envisaged to identify region/pockets where the target species habitat suitability was maximum, moderate, low or least. Further, we intended to identify, how the present distribution would be affected in the face of climate change using different climate projections parameters. Also, habitat transformation (i.e., stable, gain or loss) and change analysis was conducted to depict vulnerable areas and areas which will remain suitable in the next 50 years |
| 2 | Lines 89-93. The first time that the study species is mentioned, complete information about its distribution area should be provided. As I mentioned above, the species has a quite large distribution area. Some basic information about the morphology, taxonomy, ecology across its range, and conservation status is also mandatory. | We thank the reviewer for the suggestion. The necessary information asked for is added into the manuscript. |
| 3 | 2. Lines 100-101. The authors should provide the reasons why the study area is restricted to Uttarakhand. As I mentioned above, including aims related to the local conservation issues in this state could make this paper stronger. | We agree with the reviewer viewpoint regarding elsewhere reported records of the target species. The state of Uttarakhand was extensively ground truth, which in others cases would not have been possible. Further, without validating a model with the field data would be biased in true sense. |
| 4 | 3. Lines 149-150. A way to improve the paper could be to incude other GCMs (such as MIROC and others). | We thank the reviewer for his/her remark. After extensive literature review on Himalayan species distribution modelling, the present model was selected. We believe there is prediction disparity among different models in different regions. |

**## Reviewer 2**

| **S. No.** | **Comments/suggestions/ queries** | **Response** |
| --- | --- | --- |
| 1 | L36-38 I suggest removing all the Bio, and putting directly the variable name that is in parentheses. | We thank the reviewer for bringing up this correction. Needful actions has been taken in the revised manuscript |
| 2 | L43-44 All these regions mentioned here should appear on the map in Figure 1. For readers, like me, who do not know the study region, they will not be able to identify and understand the results without this information in the figure. | Map of the Figure 1 has been revised extensively, with the region having high habitat suitability marked. |
| 3 | L44-46 Considering that the title of the paper states that the study is about the evaluation of an endangered medicinal species, the conclusion of the paper should have this approach. From the title of the paper, the readers will look in the abstract for the conclusion of the study - what was found looking forward? The current conclusion is quite vague. | We thank the reviewer for his/her constructive remark. In the revised manuscript, abstract conclusion has been made more conclusive and drawn factual statement. |
| 4 | L48-49 These keywords (Dactylorhiza hatagirea, Habitat distribution [space], Climate change) are already in the title. It is best to use different words | We thank the reviewer for his/her suggestions. Necessary actions has been undertaken and revised |
| 5 | L52 Climate is an… | Incorporated as suggested |
| 6 | L63 Remove or inform which are the “etc.” | In the revised manuscript “etc” has been removed as suggested |
| 7 | L71 Remove or inform which are the “etc.” |  |
| 8 | L74-75 I consider that before this paragraph, another paragraph with the main approach of the current manuscript needs to be developed. What are the future predictions for mountainous areas in the region? Mountain environments are quite sensitive to climate change, and small changes are already enough to alter the habitat suitability of species. I think the authors need to address this a bit, as well as changes in environmental conditions from climate change across elevational gradients. What are the expected impacts, and already known to science? What are the predicted or potential impacts on the species niche if changes occur? | We thank the reviewer for the suggestions. In the revised manuscript, a separate paragraph has been added to address all the suggestion raised by the reviewer. |
| 9 | L100-104 Please add question marks to the questions. | Necessary action is undertaken |
| 10 | L113-116 What is the range of altitude? | In the Figure 1, altitudinal class across different region is provided, so as to depict highland and lowland areas within the state boundary |
| 11 | L258-262 Again, it is not possible to identify these regions in this figure. | The regions mentioned in the manuscript has been outlined with respective boundary files and respectively labeled |
| 12 | L328-331 All this information is already available in the results. It is repetitive. | The repetitive text has been deleted |
| 13 | L421 The title of some REF are written in uppercase and others in lowercase. Standardize all of them to lowercase. Also, some REFs (4, 10, 43) are missing spaces between their names. Review REF 30. | Necessary actions has been undertaken and revised |
| 14 | I suggest that the authors revise the lack of space between words, because this occurred throughout the text (e.g., L41, L346, L354,....) | Necessary actions has been undertaken and revised |
| 15 | As the maps provided have low resolution, it was not possible to visualize the change of patterns for the different scenarios according to the changes in coloration. | We support the reviewer viewpoint in the revised manuscript; the resolution of the maps has been increased. Also, change-shift maps has been divided into separate maps of each, so as to make them more visible and meaningful |
| 16 | Anyway, besides increasing the font size and resolution of these figures, I suggest that the authors put the name of the axis with the biological variable (e.g., minimum temperature), and not the code given by WorldClim (e.g., Bio 2). But this code could come in parentheses. | We thank the reviewer for the suggestion, the highlighted figures has been revised. |
|  |  |  |
