# Supplementary material for: Predicting the potential distribution of Dactylorhiza hatagirea (D. Don) Soo-an important medicinal orchid in the West Himalaya, under multiple climate change scenarios
Source: PLoS One. 2022 Jun 17;17(6):e0269673. doi: 10.1371/journal.pone.0269673 (PMC9205508; doi:10.1371/journal.pone.0269673)
Supplement: Attachment — Submitted filename: Response to Reviewers Comments_2nd revision.docx [file pone.0269673.s005.docx]

**Reviewer # 3**

| **S. No.** | **Comments/suggestions/ queries** | **Response** |
| --- | --- | --- |
| 1 | I believe that the most significant problem in the language!  Although I am not a native English speaker, I feel that the manuscript would be greatly improved if it would be checked by someone with excellent skills in the English language. | We thank the reviewer for the needful revision; in the revised manuscript, grammatical errors and other languages shortfalls has been significantly improved |
| 2 | P.3, L.56: “ ……. and abiotic factor affecting species potential geographic…..” | Revised as suggested (L. 56-57) |
| 3 | P.3, L.71: “….. is much higher than the global average”. Delete the word “rate”. | We thank the reviewer for the suggestion. Needful revisions are undertaken in the manuscript (L. 71) |
| 4 | P.4, L. 83: “….. with 31% of them being native, whereas 15.5 % endemic and threatened.” | Revised as suggested (L. 82-83) |
| 5 | P.4, L. 85: “Other challenges …..” | We thank the reviewer for his/her remark. Needful revision is made in the manuscript (L. 84) |
| 6 | P.4, L.97: “Therefore, estimation current plants distribution and identifying important climatic refugia will help predict future distribution …..” | Needful revision is undertaken in the revised manuscript (L. 96) |
| 7 | P.4, L.99: ecological niche modelling is a set of different techniques, whereas Maxent is just one of them. So, these two terms should not be treated as the same. In your case I would suggest to make a small introduction to the SDMs in general, and afterwards you could write one or two sentences about Maxent. | We thank the reviewer for the needful correction. The revised manuscript differentiates between MaxEnt and other simulation tools. Also, as suggested, a small introduction about SDM is added.  (L. 99-104) |
| 8 | P.5, L. 101. “Using these input variables”. Which variables? You did not mention any kind of variables. You should say “Using environmental variables/predictors …”. | We thank the reviewer for his/her remark.  Needful corrections are undertaken  (L. 104-105) |
| 9 | P5-6, L.105-127: I suggest stating other authors (Thakur et al. 2021; Shrestha et al. 2021) who predicted current and future potential distribution of D. hatagirea and after that you can add a part about the novelty of your study. | With reference to Comment No. 31 (Sl. No), we have briefly discussed the finding of both authors and integrated them with the present study findings  (L. 352-472) |
| 10 | P.6, L.140: “…. harbors alpine vegetation, which covers …..” | Revised as suggested (L. 145) |
| 11 | P.6, L. 141: Replace the phrase “in totality it forms about” with “corresponding to c. 24.11%” | Revised as suggested (L. 146) |
| 12 | P.6, L.142: Either write “The alpine areas are well known for their high-value …” or “The alpine area is well known for its high-value ….” | Revised as suggested (L. 147) |
| 13 | P.7, L. 147: “presence points” instead of “presence point” | We thank the reviewer for the remark; needful correction is made in the revised manuscript (L. 152) |
| 14 | P7, L.150-151: “Data on the species were very limited, whereas herbarium records were not geo-referenced.” | Needful correction is made  (L. 155-156) |
| 15 | P.7, L. 152: “presence points were recorded.” | Revised as suggested (L. 157) |
| 16 | P.7, L.155: “decimal degrees” instead of “degrees decimal” | We thank the reviewer for his/her remark; needful revision is made in the revised manuscript (L. 160) |
| 17 | P.8, L.168: can you please say a few words why you used CCSM4 instead of others? | After extensive literature review and synthesis from the studies (mainly from the Hindu-Kush Himalayan region), we needed a balance model to fulfill our objective |
| 18 | P.8, L. 172: “edaphic properties are expected to remain stable” | Revised as suggested (L. 176-177) |
| 19 | P.8, L. 173: “hence the same raster layer was used in future projections.” | Revised as suggested (L. 178) |
| 20 | P.9, L.178-189. This part should be rewritten and should be checked by a native English speaker. | We thank the reviewer for the needful revision; this section has been revised to develop a meaningful sentence.  (L. 182-191) |
| 21 | P.10, L. 232. I wouldn’t say that reduction in the number of predictors increases the predictive power of the model. Instead of that, if you use highly inter-correlated variables, then you will not be able to identify those that are highly important for the distribution of the studied species. And this is owned to the high correlation coefficient between different predictors. | We thank the reviewer for the suggestion, and we are in line with/ agree with the reviewer's viewpoints. The sentence in the revised manuscript has been rephrased  (L. 243-246) |
| 22 | Authors used AUC to assess the predictive accuracy of their model. Actually, I didn’t read that they used the average model prediction made after 30 runs. However, high AUC values, as in the case of this study, may be owned to the low number of species records. Instead of using AUC value, I would recommend of running the model a number of times (e.g. 10 or 30 runs) and then select the best model by using the Akaike information criterion (AICc) (Warren & Seifert 2011). | We thank the reviewer for his/her remark. The needful revision sought is revised.  In the revised manuscript, other than relying on AUC for assessing the model accuracy, we also calculated sensitivity, specificity, and true skill statistics (TSS) (Lobo et al. 2008; [https://doi.org/10.1111/j.1466-8238.2007.00358.x](https://doi.org/10.1111/j.1466-8238.2007.00358.x)), which are widely used to access the model accuracy.  (L. 220-229 & L. 254-259) |
| 23 | P. 13. L. 272: “The TIC value is the habitats suitability class of D. hatagirea on the maxent model.” What do you mean? Please rephrase that sentence. | We thank the reviewer for the needful revision; the sentence has been deleted as the conviction made failed to make any fruitful argument in the preceding paragraph (L. 289-90) |
| 24 | In several parts of the manuscript, a semi-colon (;) was used instead of a comma. | The manuscript has been thoroughly revised with all the parameters in consideration of the present comments |
| 25 | p.17, L. 337: Authors used the term “Habitat distribution modelling”. This term is used here for the first time, instead of others that were used in other parts of the manuscript. I would suggest using the same terminology throughout the text | We thank the reviewer for his/her remark. In the revised manuscript, for the shake of uniformity, SDM is used throughout the manuscript |
| 26 | p. 17, L.340: “under a changing climate scenario” | Revised as suggested (L. 360) |
| 27 | p.17, L.341-342: SDMs provide the potential distribution. However, species distributions are determined both by abiotic factors and biotic interactions. Orchids are characterized by strong biotic interactions which have to do with the mycorrhizal fungi that will help them germinate and keep feeding them, as well as by specific insects which will be their pollinators. Such biotic interactions are very important and, in many cases, can influence orchids’ potential distribution. Such limitations should be mentioned in the manuscript. You can read Evans et al (2021) and Tsiftsis & Djordjevic (2020). | We thank the reviewer for the needful revision. The needful suggestion has been incorporated, and limitations of the study are adequately mentioned  (L. 466-473) |
| 28 | P.18, L.349-350: I think that the term “species density” is wrong. Can you rewrite that part as this term is referring to a number of different species? | We thank the reviewer for the suggestion; needful action in the sentence is undertaken in the revised manuscript  (L. 409-412) |
| 29 | P.18, L. 354: add a comma after the word “variables”. | Revised as suggested |
| 30 | p.18, L.355-358: please rewrite that part. It is not clear what you want to say! | We thank the reviewer for the needful revision. This section has been re-written and revised accordingly (L. 409-417) |
| 31 | The findings of this study should be discussed in relation to the findings of Thakur et al. (2021) and Shrestha et al. (2021), which at this stage are ignored. | We thank the reviewer for the suggestion. In the discussion section, both the publication and other studies conducted on the target species are adequately discussed with the present study's finding. (L. 353-373) |

**## Reviewer- 4**

| **S. No.** | **Comments/suggestions/ queries** | **Response** |
| --- | --- | --- |
| 1 | L32. With these models, the authors are not determining the future geographical distribution, but the suitable areas (in terms of environmental variables). | We thank the reviewer for bringing up this correction. Needful actions have been undertaken and revised accordingly (L. 32-34) |
| 2 | L41. Most of the species loss habitat suitability in future scenarios. Especially when they move to higher altitude which by the conic shape of mountains, area is less available. Of course this is possible if species may disperse to different areas where is found in contemporary habitat. Add in what percentage is expected to expand. | We thank the reviewer for the suggestion; needful expansion percent has been incorporated in the revised manuscript (L. 40-42) |
| 3 | L59. Not necessarily. Some species are more capable than others, not all species will show the same responses. | We partially agree with the reviewer's viewpoint. Yes, all species may not reflect the same response, but here, we intend to emphasize on those species (especially in the context of the Himalayan region) where such responses are adequately seen. Such responses are often among the Rare, Endangered, and Threatened (RET) species in the Himalayan region. Although we still believe more conclusive needs to be conducted to strengthen this argument elsewhere and in the Himalayas.  (Manish, 2022; <https://doi.org/10.1016/j.ecoinf.2021.101546>)  Rather et al. (2022) (<https://doi.org/10.1016/j.ecoleng.2021.106534>)  Hamid et al. (2018) (https://doi.org/10.1007/s10531-018-1641-8) |
| 4 | L69. The increased in greenhouse gases emission is not only for the region, but for the entire planet. | We agree with the reviewer's viewpoint, but if we compare the impacts of climate change across the globe and the Himalayas in several reports, including IPPC and several other projection reports, the cascading impacts of climate change in this region are just double than that of the global average.  <https://report.ipcc.ch/ar6wg2/pdf/IPCC_AR6_WGII_CrossChapterPaper5.pdf>  Sabin et al. (2020) (<https://doi.org/10.1007/978-981-15-4327-2_11>)  Liu J, Rasul G. Climate change, the Himalayan mountains, and ICIMOD. Sustainable mountain development. 2007; 53:11-4.  Rafiq et al. (2022) (https://doi.org/10.1007/978-3-030-89308-8_12) |
| 5 | L106. The species is an orchid, right? Authors should say this here. | For kind consideration and also for better understanding of the readers, the identity of the species is added in the Title of the manuscript itself (L. 1-2; L. 29) |
| 6 | L116. Not clear what do the authors mean with “the species require…..conditions for growth and perturbation….” | Other than being an endemic (i.e., Himalaya), the target species also have high habitat preferentially towards a particular set of conditions. In this region, local microclimatic conditions play an important role in shaping or determining the extent of such species' distribution.  The sentence has been re-phrased for better understanding (L. 118-119) |
| 7 | L118. “The existing multiplication for mass multiplication” sounds odd to me. | We thank the reviewer for highlighting this; the sentence has been re-phrased in the revised manuscript (L. 119-122) |
| 8 | L121. Delete “present” | Revised as suggested (L. 122-123) |
| 9 | I´d suggest a section for Study species with a brief taxonomic description and more on the use and management. I´d include detailed pictures of the species (flowers, leaves, bulbs), individuals in the wild and also pictures of the products in trade. | We thank the reviewer for his/her remarks. In the introduction, a brief taxonomic description of characteristics is added. (L. 110-112)  A separate Figure 1 is added in the revised manuscript to address the follow-up question. (L. 131-132) |
| 10 | Is the species included in the IUCN Red List? In case is not I´d highly recommend to make the IUCN assessment following the IUCN criteria and include this as part of results? | As per IUCN 2022 (<https://www.iucnredlist.org/>) the species is not included in the threatened category; however, at the regional level, the species is categorized as ‘Critically Endangered’ (CAMP; <http://envis.frlht.org/camp.php>).  As the species is not included in IUCN 2022 threat category, the Title of the manuscript is revised (L. 1-2).  In the subsequent publication, the threat assessment as per the IUCN criteria would be undertaken |
| 11 | 2.3. Climate data. I suggest a more general subtitle for this subsection. What the authors are using is not only climate data. They are using geomorphologic, pedologic and LULC. | We thank the reviewer for highlighting this. A general title as ‘Data source’ is included in the revised manuscript for better understanding (L.162) |
| 12 | L212. AUC was the only statistical test used assess the models? Since several years ago, AUC has been demonstrated its reliability as a comparative measure of accuracy. I suggest to include some other tests, (for example the binomial test, Partial ROC, Trus skill statistics). Lobo et al. 2008 (<https://doi.org/10.1111/j.1466-8238.2007.00358.x>) is a must read article for this. | We thank the reviewer for the suggestion and agree with his/her viewpoint. Following this, in the revised manuscript, other than relying on AUC for assessing the model accuracy; we also calculated sensitivity, specificity, and true skill statistics (TSS) using confusion matrix preparation according to Alloche et al. (2006) (doi: 10.1111/j.1365-2664.2006.01214.x)  (L. 220-229 & L. 254-259) |
| 13 | L217. The used classification especially for comparison among RCPs and periods (Table 4; Figure 5, Figure 7 and 8) is quite confusing at least to me. I would use instead a binary classification (0/1, suitable/unsuitable) using only one threshold. This way would be much more clear to compare among RCPs and periods (see suggestions in the Results section). | We thank the reviewer for the suggestion. As stated in Comments No. 6 (Sl. No) regarding high habitat preferentiality and contagious distribution (mostly) of the species, here we aimed to elucidate and identify pockets that are most, moderate, low, and least suitable habitat in the given study area. Doing this, it is envisaged to provide a clear-cut picture of where the species can be translocated or habitats that can be taken up for medicinal plant conservation and developmental area.  As Figures 7 & 8 giveaway the same information as given in Table 4, therefore in the revised manuscript, both the Figures are added as supplementary Figures 1 & 2. (L. 325)  The habitat suitability classification used in this study was prepared following the methodology of Adhikari et al. (2012) (doi:10.1016/j.ecoleng.2011.12.004), Yang et al. (2013)(<http://dx.doi.org/10.1016/j.ecoleng.2012.12.004>), and Zhang et al. (2019) (<https://doi.org/10.1016/j.ecoinf.2019.01.004>);  Arslan et al. (2020) (https://doi.org/10.1007/s10113-020-01695-6) |
| 14 | L235. Table 1 is in the Methods section, but it is a Result. I suggest to include it only here and not in Methods. | We thank the reviewer for the suggestion; needful revision is incorporated in the revised manuscript  (L. 247) |
| 15 | Table 1. I see the information on this table as a result. Why is it in methods? |  |
| 16 | Table 3. It is not clear to me how the Gain, Loss, Stable and Unsuitable areas were computed. Authors did use a classification of Unsuitalbe, low suitable, moderate suitable | The geographical distribution of the species in terms of unsuitable, low-moderate suitable, and high suitability was initially determined using the classification of Adhikari et al. (2012) (doi:10.1016/j.ecoleng.2011.12.004), Yang et al. (2013)(<http://dx.doi.org/10.1016/j.ecoleng.2012.12.004>), and Zhang et al. (2019) (<https://doi.org/10.1016/j.ecoinf.2019.01.004>);  Arslan et al. (2020) (https://doi.org/10.1007/s10113-020-01695-6)  Subsequent to this, to determine gain, loss, or stable habitats, we took the pixels value of the current distribution (of the above-classified habitats) and subtracted them with the pixels values of the classified habitats of the two climatic scenarios (i.e., 2050 & 2070).  A similar study is undertaken in some of the previous studies:  Coban et al. (2010) Investigation on changes in complex vegetation coverage using multi-temporal landsat data of Western Black sea region-a case study. J Environ Biol 31:169–178  Arslan et al. (2020): https://doi.org/10.1007/s10113-020-01695-6 |
| 17 | Table 4. Are the authors presenting the same information in Table 4 than in the Maps (figure 7 and 8)? Highly recommended to include the information only once. | We thank the reviewer for the suggestion, as Fig. 7 & Fig. 8 represent the same information as in Table 4; therefore, we decided to add both the Figures as supplementary figures in the revised manuscript  (L. 325) |
| 18 | Fig. 1. I suggest to add (at least in a small inset) the Map of India. | Figure 1 has been revised as per suggestion, and now in the revised manuscript, Figure 1 is marked as Figure 2 due addition of a new Figure as Figure 1 in its place |
| 19 | Figures 2-4 are those generated by default by Maxent. I don´t see these figures essential to be included in the results. They could not be included or maybe only as supplementary. | We thank the reviewer for the suggestions, but we believe retaining both the Figures might be helpful for knowledgeable readers and understanding parameters having a larger influence on the species distribution |
| 20 | Figures 7 and 8 and the colors used are not useful to explore what is the future of the species. The main problem from my point of view is the 11 categories resulting of comparisons between RCPs and years (2050 and 2070). I would not use this comparison. I would do the comparison using the gain, loss, stable and unsuitable categories, considering only the binary classification. | We thank the reviewer for the suggestion, as Fig.7 and Fig.8 represent the same information as mentioned in Table 4; therefore, both the Figures are now added as supplementary datasets.  Yes, using 11 color categories can be quite confusing, but at the same time, our aim of the study was to pinpoint areas that are expected to see transformation in their habitat using the climate projection model for better advocacy of the management plan. |
|  |  |  |
